# Retinal Ganglion Cell Degeneration in Glaucoma: Systematic Review

**DOI:** 10.3390/bioengineering13050509

**Published:** 2026-04-28

**Authors:** Masuma Firoz, Neloy Shome, Noah Wong, Prisha Jonnalagadda, Hari Tunga, Amirmohammad Shafiee, Amirmahdi Shafiee, Sohan Bobba, Karanjit S. Kooner

**Affiliations:** 1 Department of Ophthalmology, University of Texas Southwestern Medical Center, Dallas, TX 75390, USA; masumafiroz99@gmail.com (M.F.); nshome825@gmail.com (N.S.); noah.wong@ttuhsc.edu (N.W.); prisha.jonnalagadda@utsouthwestern.edu (P.J.); haristunga@gmail.com (H.T.); a.shafiee@tcu.edu (A.S.); amirmahdishafiee07@gmail.com (A.S.); sohanbobba@gmail.com (S.B.); 2 Long School of Medicine, University of Texas Health Science Center at San Antonio, San Antonio, TX 78229, USA; 3 School of Natural Sciences and Mathematics, University of Texas at Dallas, Richardson, TX 75080, USA; 4 Burnett School of Medicine, Texas Christian University, Fort Worth, TX 76104, USA; 5 Department of Ophthalmology, Veteran Affairs North Texas Health Care System Medical Center, Dallas, TX 75216, USA

**Keywords:** retinal ganglion cells, glaucoma, neurodegeneration, neuroprotection, neuroregeneration, artificial intelligence

## Abstract

Retinal ganglion cell (RGC) degeneration underlies glaucomatous optic neuropathy and remains a leading cause of irreversible vision loss worldwide. Although elevated intraocular pressure (IOP) is the primary modifiable risk factor, RGC death reflects converging mechanisms including mechanical stress, vascular insufficiency, metabolic dysfunction, and neuroinflammation. We conducted a PRISMA-guided systematic review with PICOS-defined eligibility criteria, searching PubMed, Cochrane Library, ScienceDirect, Scopus, Google Scholar, and ProQuest for studies through January 2026 on RGC degeneration and neuroprotective or regenerative therapies in glaucoma. Included studies supported OCT-based structural assessment and imaging biomarkers as essential tools for early detection, risk stratification, and monitoring of progression and treatment response. Continued RGC loss despite IOP control in many patients highlights the need for mechanism-based interventions; neuroprotective strategies targeting excitotoxicity, oxidative stress, mitochondrial dysfunction, and neurotrophic insufficiency are emerging, while stem cell and gene-based regenerative therapies remain under active investigation. Integrating molecular insights with advanced imaging and biomarker-guided endpoints may enable earlier, more individualized intervention and help explain progression despite adequate pressure control.

## 1. Introduction

Glaucoma is a group of ocular conditions that often lead to elevated intraocular pressure (IOP), which causes progressive degeneration of the optic nerve head (ONH), resulting in corresponding visual field (VF) defects [1]. Well-known risk factors include older age, family history of glaucoma, hypertension, diabetes, and obstructive sleep apnea [1]. Glaucomatous optic neuropathy (GON) is the leading cause of irreversible blindness, affecting more than 80 million individuals worldwide, including approximately 4.2 million in the United States as of 2024. These figures are projected to rise to 180 million globally and 11.3 million in the United States by 2060 [2].

**Figure 1:**
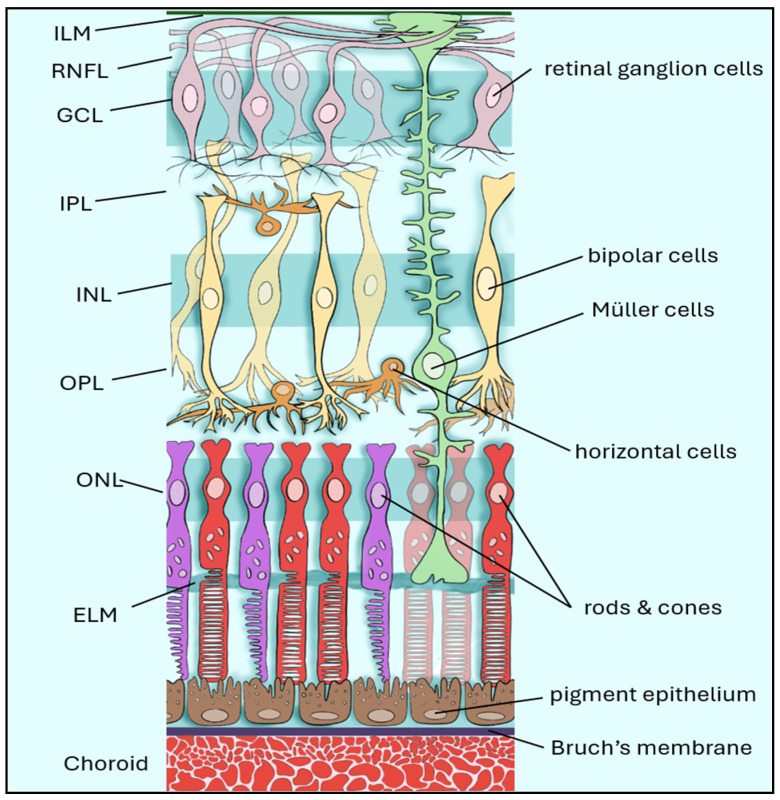
Layers of the retina. Retinal ganglion cells reside in the second innermost layer of the retina, and their axons converge to form the retinal nerve fiber layer and subsequently the optic nerve. Adapted from [3]. **Abbreviations**: inner limiting membrane (ILM), retinal nerve fiber layer (RNFL), ganglion cell layer (GCL), inner plexiform layer (IPL), inner nuclear layer (INL), outer plexiform layer (OPL), outer nuclear layer (ONL), and external limiting membrane (ELM).

**Figure 2:**
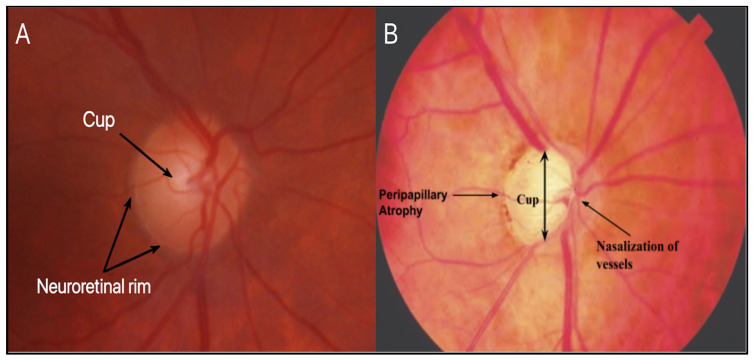
(**A**) Fundus photograph of a healthy person: cup-to-disk ratio of 0.2 and normal neuroretinal rim. (**B**) Features of glaucomatous optic neuropathy: cup-to-disk ratio of 0.95, nasalization of vessels, and peripapillary atrophy.

Retinal ganglion cells (RGCs) reside in the inner retina, with their axons forming the retinal nerve fiber layer (RNFL) as they converge toward the optic nerve head (Figure 1) [3]. The underlying pathology of glaucoma is characterized by progressive damage to RGCs and their axons, leading to structural and functional deterioration of the optic nerve (Figure 2B). This process, termed glaucomatous optic neuropathy, is multifactorial in nature and reflects the interplay of biomechanical, vascular, and molecular factors contributing to RGC injury. Importantly, trabecular meshwork dysfunction represents an early event in this process, connecting anterior segment pathology with downstream RGC injury [4]. Furthermore, acellularity of the TM has been recognized as one of the earliest signs of glaucoma, interfering with aqueous humor outflow and leading to elevated IOP [4].

**Figure 3:**
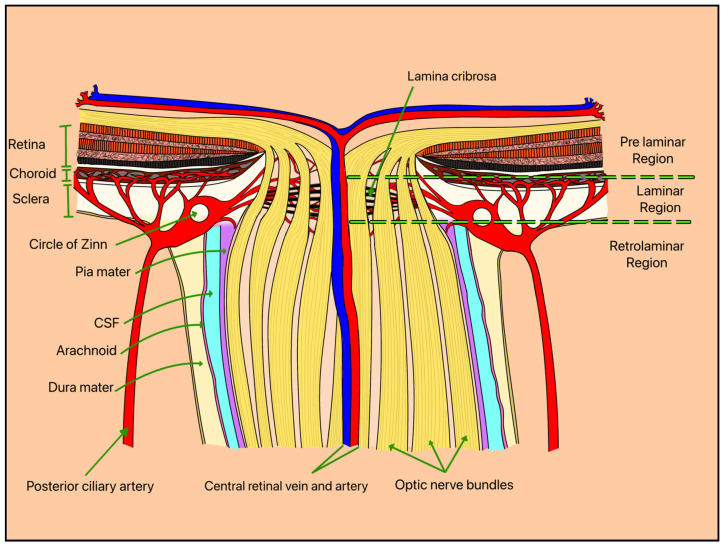
Diagram showing the components of the optic nerve (prelaminar, laminar, and postlaminar regions, nerve fiber bundles, central retinal artery and vein, and posterior ciliary arteries including the Circle of Zinn). Adapted from [6]. **Abbreviations**: CSF: cerebrospinal fluid.

**Figure 4:**
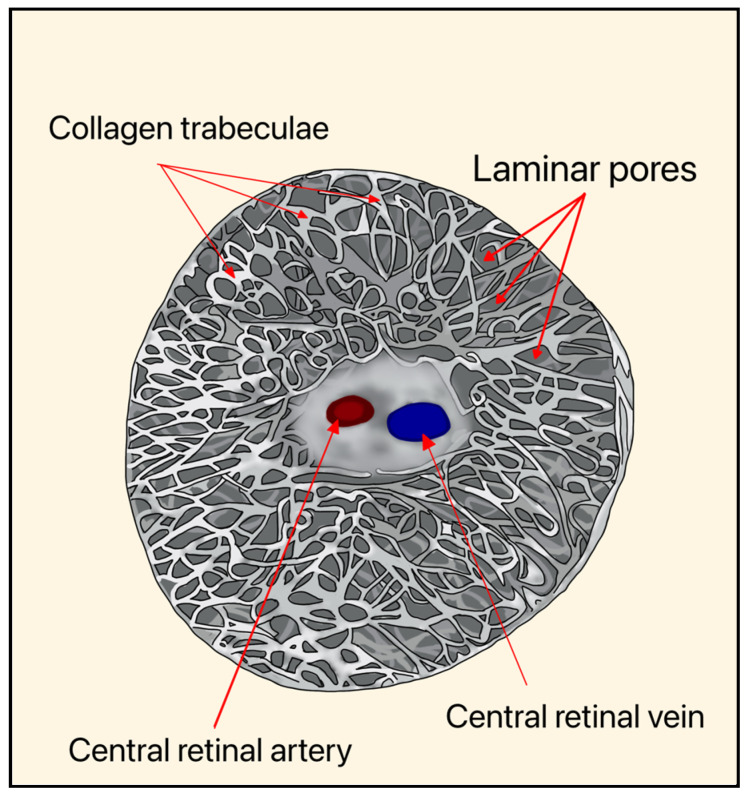
Cross-sectional view of the lamina cribrosa, highlighting the structural region through which retinal ganglion cell axons pass and are subjected to biomechanical stress. Adapted from [6].

Elevated IOP and vascular dysregulation represent two central, interconnected mechanisms underlying RGC degeneration in glaucoma. Biomechanical stress is thought to occur primarily at the level of the optic nerve head (ONH), where RGC axons pass through the lamina cribrosa (LC), a structurally vulnerable region susceptible to pressure-induced deformation (Figure 3 and Figure 4). This strain may disrupt axonal transport and contribute to progressive neuronal injury [5].

**Figure 5:**
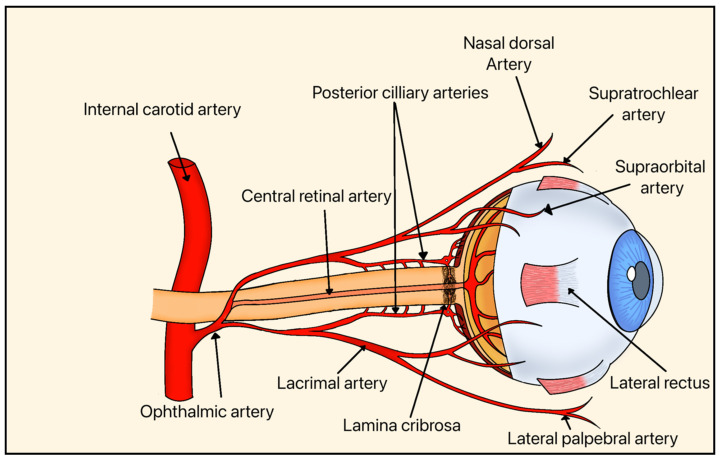
Ocular blood supply. The central retinal artery and posterior ciliary arteries provide critical perfusion to retinal ganglion cells and their axons, particularly at the level of the lamina cribrosa. Adapted from [8].

Systemic vascular factors further contribute to RGC injury through impaired perfusion of the ONH and inner retina, leading to ischemia and metabolic stress. The ONH and inner retina, where RGCs reside, are supplied primarily by branches of the central retinal artery and the short posterior ciliary arteries, including the Circle of Zinn-Haller (Figure 5). This vascular network is critical for maintaining oxygen and nutrient delivery to RGC axons, particularly at the level of the LC, where structural and metabolic demands converge. Reduced ocular blood flow and dysregulation of autoregulatory mechanisms have been implicated in glaucomatous progression [7].

Rather than representing competing explanations, biomechanical and vascular mechanisms are increasingly recognized as interdependent processes. Elevated IOP may induce both structural deformation and secondary vascular compromise, resulting in combined mechanical and metabolic stress on RGCs. In patients with optic nerve vascular insufficiency without elevated daytime IOP (normal-tension glaucoma), nocturnal ocular hypertension may still contribute to GON [1,7,9]. These overlapping insults act synergistically to drive progressive neurodegeneration. While both mechanisms are supported by experimental and clinical evidence, their relative contributions likely vary across individuals, reflecting the multifactorial nature of glaucoma [1,7,9,10].

Whether through direct mechanical compression, vascular compromise, or a combination of both, RGC death and axonal degeneration represent the final common pathway leading to irreversible vision loss in glaucoma. Understanding the central role of RGCs in glaucoma is essential for advancing diagnostic tools and developing targeted therapies; however, current knowledge remains fragmented across molecular mechanisms, imaging biomarkers, and emerging therapeutic strategies. While prior reviews have examined these domains in isolation, an integrated framework linking pathophysiology with diagnostic and therapeutic advances remains lacking.

Therefore, this systematic review aims to synthesize current evidence on RGC degeneration in glaucoma within a unified conceptual model that connects structural, vascular, and molecular processes with disease progression, artificial intelligence monitoring, and clinical solutions. In summary, we examine how converging mechanisms drive RGC loss and how this integrated understanding can inform the development of improved diagnostic strategies and targeted therapeutic approaches.

## 2. Materials and Methods

### 2.1. Initial Search (Figure 1)

**Figure 6:**
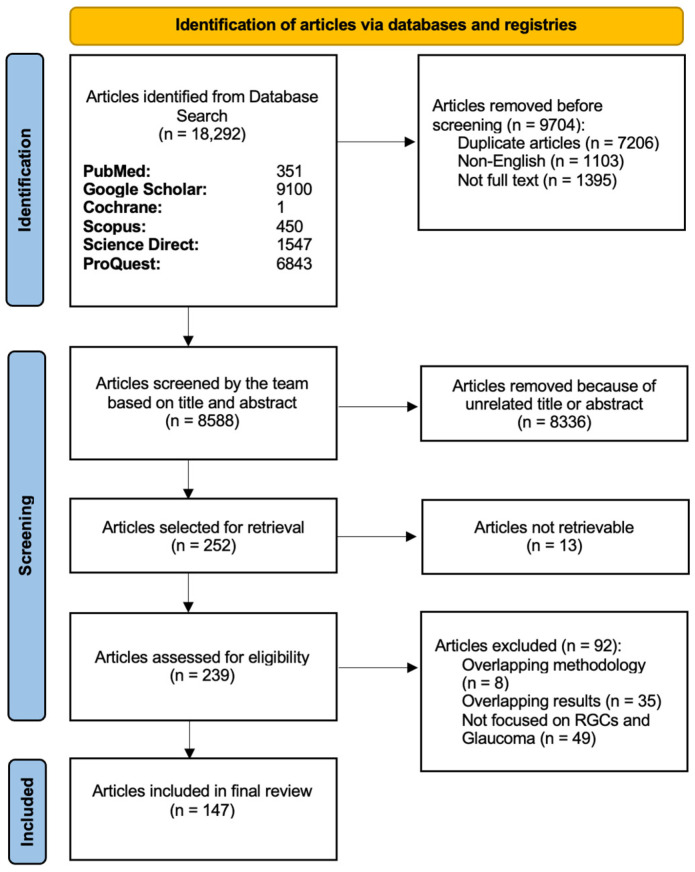
PRISMA flowchart depicting manuscript identification and selection process.

We followed the standards outlined by Preferred Reporting Items for Systematic Reviews and Meta-Analyses (PRISMA) guidelines (www.prisma-statement.org) during data collection (Figure 6). The following keywords and MeSH terms were selected: (retinal ganglion cells) or (retinal ganglion cell) or (RGC) and (glaucoma) and (cell death) or (apoptosis) and (neuroprotective agent) or (neuroprotective properties) or (regenerative properties) or (regenerative agents). Using these keywords and MeSH terms, we systematically searched the online databases of PubMed (MEDLINE), Cochrane Library, ScienceDirect, Scopus, Google Scholar, and ProQuest up to January 2026. The results from the different databases were downloaded and saved as a comma-separated values (CSV) file and then compiled together on Google Sheets. We created a customized Python (Python Software Foundation, Wilmington, DE, USA, version 3.9.23) based script and downloaded all the references from Google Scholar in one file.

### 2.2. Protocol Registration

The review was registered in PROSPERO (International Register of Systematic Reviews; registration number CRD420261278717). The scope of the review was refined during protocol development to align with emerging advances in RGC classification and therapeutic strategies relevant to glaucoma.

### 2.3. Preliminary Screening

The fully compiled list from Google Sheets was then input into Rayyan AI (Rayyan Systems Inc., https://www.rayyan.ai/, Cambridge, MA, USA), a web-based platform. The Rayyan AI platform was only used as a tool for storing the articles gathered during the systematic review and efficiently performing manual screening of the articles. The preliminary exclusion criteria consisted of non-English articles and study types such as conference abstracts, commentary, and duplicate papers with the same digital object identifier (DOI).

### 2.4. Eligibility Assessment

The PICOS (population, intervention, comparison, outcomes, and study) framework (https://libguides.mssm.edu/ebm/ebp_pico, accessed on 15 April 2026) was used to create eligibility criteria (Table 1). The remaining manuscripts were screened (KK, HT, NS, and MF) for eligibility assessment based on the title and abstract. Studies were included if they investigated RGC structure, function, degeneration, or therapeutic targeting in the context of glaucoma or related optic neuropathies. Eligible studies included experimental (in vitro and in vivo), clinical, and translational research articles evaluating molecular mechanisms, imaging biomarkers, or therapeutic interventions relevant to RGC health. Studies were excluded if they were non-original articles (e.g., editorials, commentaries), non-English, lacked relevance to RGC biology or glaucoma, or did not provide mechanistic, diagnostic, or therapeutic insight. Data extraction was performed independently by multiple reviewers as described above. Extracted information was recorded and organized in a structured format for qualitative synthesis. Discrepancies were resolved through discussion among the study authors, and blinding was utilized through the Rayyan AI platform during study selection. For each included study, the following data items were extracted: publication year, study design (clinical, preclinical, or etiological), model system (human, animal, or in vitro), country of origin, and key findings related to RGC structure, function, or degeneration. Additional data included reported molecular mechanisms (e.g., excitotoxicity, oxidative stress, mitochondrial dysfunction, neuroinflammation), imaging biomarkers (e.g., OCT(A), RNFL thickness), and therapeutic strategies targeting neuroprotection or regeneration. Detailed exclusions and justifications are shown in Figure 6.

### 2.5. Risk of Bias, Reporting Bias, and Certainty Assessment

Because this review included a mix of clinical, pre-clinical, basic science, and animal studies in addition to etiological studies, a formal risk-of-bias assessment (e.g., Cochrane ROB 2 or ROBINS-I) was not feasible. Instead, methodological quality was qualitatively evaluated based on study design, clarity of outcome reporting, and reproducibility of experimental methods. Reporting bias was not formally assessed, given the theoretical nature of many of the included studies, which often focused on hypothetical interactions rather than clinical outcomes. Similarly, certainty assessment of the evidence was not applicable to this review, as quantitative pooling or grading of comparative results was not performed.

## 3. Results

### 3.1. Structure, Function, and Classification of RGC Subtypes

**Figure 7:**
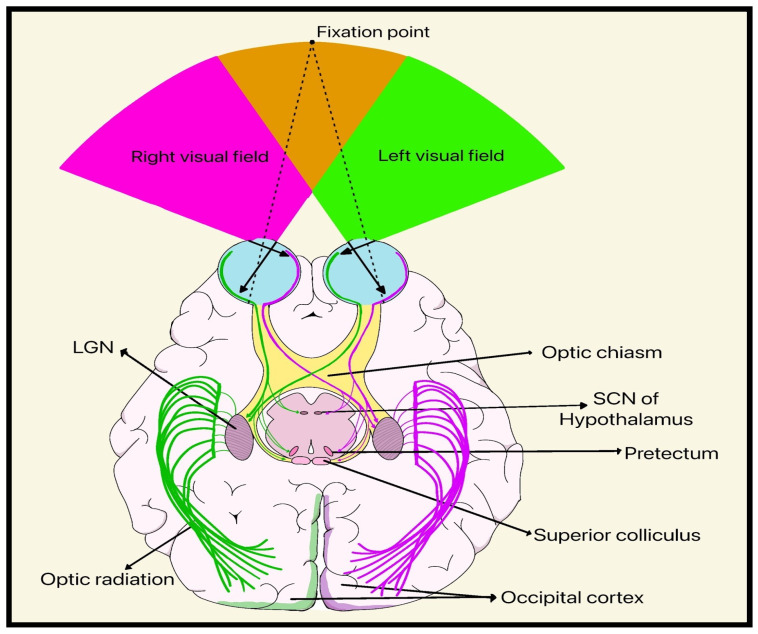
Diagram of the visual pathway showing the retina, optic nerve, optic chiasm, lateral geniculate nucleus (LGN), suprachiasmatic nucleus (SCN) of the hypothalamus, optic radiations, and occipital cortices. Original creation by the authors.

RGCs consist of a cell body, an axon, and a complex dendritic structure that receives inputs from other intermediary neurons, including bipolar cells and amacrine cells (Figure 1). The visual cascade begins when light is absorbed by photoreceptor cells (rods and cones), which causes hyperpolarization and signal transduction through bipolar, horizontal, and amacrine interneurons before reaching the RGCs. These processed signals are then relayed through the ON to the lateral geniculate nucleus (LGN) of the thalamus, the superior colliculus, and other visual centers, transforming photoreceptor activity into neural transmission (Figure 7) [11].

Multiple aspects of vision are encoded by RGCs, including luminance, contrast, spatial detail, motion, color, and directionality [3]. Their diverse electrophysiological properties enable parallel processing of distinct visual channels, as some RGCs respond preferentially to light increments (ON-cells) or light decrements (OFF-cells) while others are tuned to temporal frequency, motion direction, or chromatic opponency [3].

Although more than 40 RGC subtypes have been identified in mammals based on morphology, physiology, and molecular expression profiles, only five are primarily involved in visual signal transmission [3]. A summary of the dominant RGC subtypes, including their function and pathway, is shown in Table 2.

While numerous RGC subtypes have been characterized based on morphology, physiology, and molecular profiles, current evidence regarding subtype-specific vulnerability in glaucoma remains limited and largely derived from experimental models. Emerging data suggest that larger, metabolically active RGCs such as parasol cells may be more susceptible to early glaucomatous injury, although findings remain inconsistent across studies [3]. This variability highlights an important gap in translating subtype-specific insights into clinically meaningful biomarkers or targeted neuroprotective strategies.

### 3.2. RGC Vulnerability in Glaucoma: Connection to Pathophysiology

RGCs are the sole output neurons of the retina, and their axons traverse a long, metabolically demanding course from the eye to central targets, placing a heavy burden on axonal transport, mitochondrial function, and energy supply. As discussed previously, the LC is an anatomically restricted region through which axons of RGCs must pass, making it a site of mechanical stress, vascular compromise, and extracellular matrix (ECM) deformation when IOP or other biomechanical insults rise [11]. Collectively, these structural and metabolic characteristics position RGCs as uniquely vulnerable to cumulative biomechanical, vascular, and metabolic stressors, supporting the concept that glaucomatous neurodegeneration reflects a multifactorial and convergent disease process rather than a single primary insult.

### 3.3. Mechanisms of RGC Damage

#### 3.3.1. Axonal Transport Disruption and Neurotrophic Factor Deprivation

Axonal transport disruption represents one of the earliest and most critical consequences of elevated IOP. Based on the mechanical theory of glaucoma, pressure-induced distortions of the LC subject RGC axons to both compressive and shearing forces [5,12]. Over time, these distortions cause ECM remodeling within the ONH, including deposition of collagen fibrils and macromolecules that narrow the laminar pores and exacerbate axonal distortion. As a result, both anterograde and retrograde movement of essential molecules through the axons becomes compromised, depriving the RGC soma of vital growth factors, metabolites, and survival signals [13].

Among these molecules, brain-derived neurotrophic factor (BDNF) is critical, as it plays a central role in RGC development, synaptic maintenance, and survival. In a rat model of OHT, RGCs treated with mesenchymal stem cells engineered to secrete BDNF demonstrated enhanced survival compared to controls [14]. Beyond BDNF, axonal transport disruption also interferes with several other key trophic and metabolic mediators, including ciliary neurotrophic factor (CNTF), nerve growth factor (NGF), and nicotinamide adenine dinucleotide (NAD^+^) and thioredoxins (Trx1/2), which are redox-regulating molecules that sustain mitochondrial integrity and combat oxidative stress [13].

Collectively, the loss of these molecules amplifies cellular vulnerability and accelerates the process of RGC degeneration. Evidence supporting axonal transport disruption as an early driver of RGC degeneration is strong, with consistent findings across experimental models [12,13,14]. However, clinical validation remains limited, and it is likely that transport failure interacts with other stressors rather than acting as an isolated mechanism.

#### 3.3.2. Excitotoxicity

**Figure 8:**
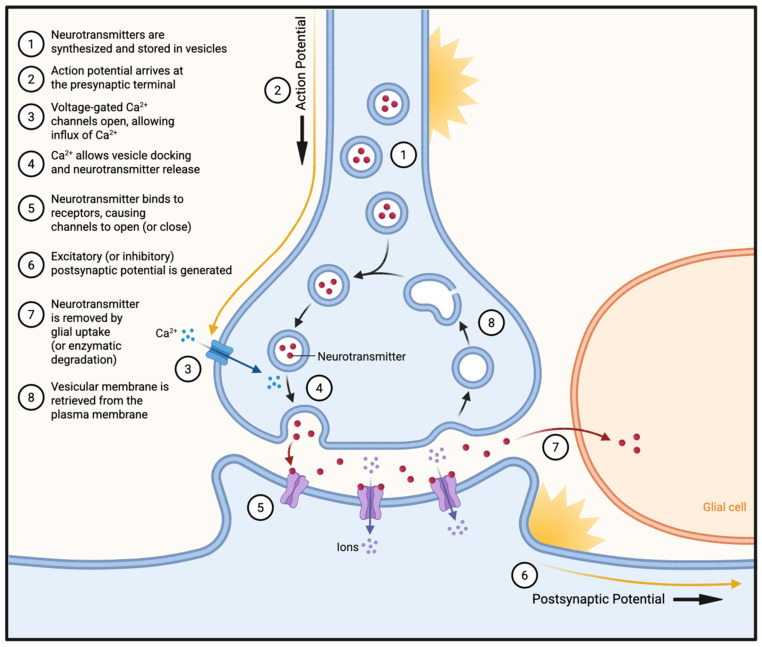
Steps of chemical neurotransmission. Glutamate receptor binding and calcium channel influx are pathologically activated in glaucoma, eventually leading to mitochondrial membrane permeabilization and apoptosis. Created in BioRender. Tunga, H. (2026) https://BioRender.com/u7l6us9.

Excessive glutamatergic stimulation leads to excitotoxic injury in RGCs when high extracellular glutamate levels activate NMDA receptors, triggering large calcium influx, mitochondrial overload, and downstream pro-death signaling (Figure 8). In glaucoma, elevated IOP, ischemia, and glial dysfunction impair glutamate uptake by Müller cells and promote excessive presynaptic release, leading to pathologic accumulation of extracellular glutamate in the inner retina. In a transgenic mouse model, intraocular injections of NMDA revealed that unique RGC subtypes demonstrated different degrees of vulnerability in response to excitotoxic stimulation, highlighting the type-specific vulnerability to glutamate-induced injury [15].

Evidence for excitotoxicity in glaucoma is moderate, with strong mechanistic support from experimental models but limited confirmation in human disease [15]. These findings suggest that excitotoxicity is unlikely to act as a uniform primary driver of RGC loss, but rather as a context-dependent and amplifying mechanism within the broader neurodegenerative process.

#### 3.3.3. Oxidative Stress

Oxidative stress in glaucoma, or the excess generation of reactive oxygen species (ROS), is caused by several factors, including older age, elevated IOP, retinal ischemia, chronic inflammation, and mitochondrial dysfunction [4]. It represents a convergent outcome of the multiple upstream insults described above. In GON, excess generation of ROS overwhelms antioxidant defenses such as superoxide dismutase, catalase, and glutathione, resulting in lipid peroxidation, protein oxidation, and mitochondrial DNA damage. These reduction and oxidation (redox) imbalances impair mitochondrial respiration, disrupt calcium homeostasis, and sensitize RGCs to apoptotic signaling. On a larger scale, oxidative damage also leads to endothelial cell decay within the trabecular meshwork, leading to altered ECM, reduced outflow facility, and ultimately elevated IOP [4]. Elevated markers of oxidative damage have been detected in both experimental models and human glaucomatous tissues, and interventions that enhance antioxidant capacity or reduce ROS accumulation have demonstrated neuroprotective effects such as reduced RGC apoptosis and preserved ON integrity [16,17].

Collectively, these findings suggest that oxidative stress is supported by robust evidence across both experimental and clinical studies, positioning it as a central convergent mechanism linking multiple upstream insults. However, targeting this mechanism in isolation may be insufficient to meaningfully alter disease progression.

#### 3.3.4. Mitochondrial Dysfunction

RGCs have exceptionally long unmyelinated axons and rely heavily on efficient mitochondrial transport and local energy production, and even subtle defects in mitochondrial dynamics or turnover can precipitate irreversible degeneration [3]. In an experimental rat model of glaucoma, researchers attempted to stabilize the mitochondrial membrane potential with hydrogen sulfide (H_2_S) in order to reverse mitochondrial dysfunction. They discovered that H_2_S supplementation prevented mitochondrial permeability transition, cytochrome c release, and downstream apoptotic signaling, preserving RGC viability [18].

**Figure 9:**
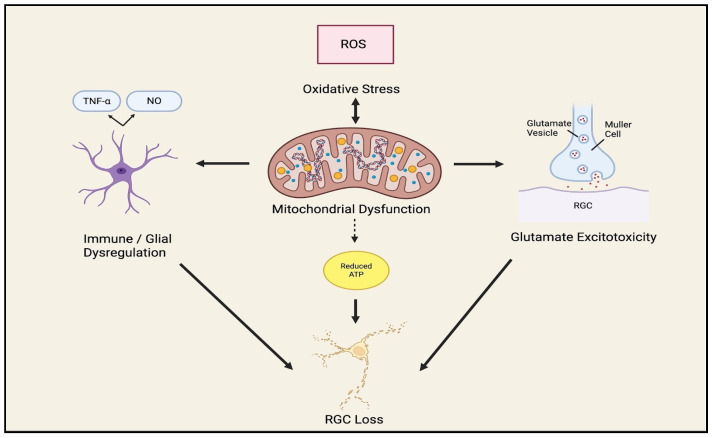
Relationships between different retinal ganglion cell stress mechanisms, such as oxidative stress, immune/glial dysregulation, glutamate excitotoxicity, and mitochondrial dysfunction. Created in BioRender. Shome, N. (2026) https://BioRender.com/5xzr7i6. **Abbreviations**: ROS: reactive oxygen species; TNF-α: tumor necrosis factor α; NO: nitric oxide; ATP: adenosine triphosphate; RGC: retinal ganglion cell.

Importantly, mitochondrial dysfunction is exacerbated by excitotoxic glutamate signaling, as calcium influx impairs mitochondrial function and leads to the generation of ROS. In turn, oxidative stress further damages mitochondrial integrity, disrupts ATP production, and amplifies vulnerability to apoptotic signaling. Mitochondrial dysfunction then exacerbates both oxidative stress and calcium dysregulation, reinforcing excitotoxic injury in a self-propagating cycle. Concurrently, these processes activate glial cells and inflammatory cascades, which further amplify neuronal stress through cytokine release and complement activation. Together, these findings suggest that mitochondrial dysfunction, oxidative stress, and excitotoxic injury act in a co-dependent cycle rather than as isolated processes (Figure 9). However, this model is supported largely by experimental and review-based evidence, with limited direct validation in human glaucoma, and the relative contribution of mitochondrial dysfunction to disease progression in vivo remains incompletely defined.

#### 3.3.5. Autoimmune Dysregulation and Glial-Mediated Neuroinflammation

Elevated IOP is thought to act as an upstream trigger by imposing mechanical and metabolic stress on the retina and ONH, thereby activating glial and downstream immune pathways, including possible T cell–mediated mechanisms, that further promote RGC injury and death [1,13,19]. This occurs because glial cells (astrocytes, Müller cells, and microglia) change from a supportive role to a reactive phenotype, resulting in further RGC injury and death [13].

A recent experimental study found that even transient elevations in IOP are sufficient to trigger retinal CD4+ T-cell infiltration, and that these T-cell responses are required for a progressive phase of RGC and axonal loss that continues even after IOP has returned to baseline. Heat shock proteins (HSPs), which are stress-response proteins, may also become targets of the immune system in glaucoma, thereby linking pressure-induced stress to autoimmune retinal injury [19]. Consistent with this, an experimental study in which mice lacked T and B lymphocytes showed less RGC loss after IOP elevation compared to immunocompetent controls, supporting a contributory role for lymphocytes in IOP-induced neurodegeneration [20]. While these studies support a role for neuroinflammation in glaucoma, the evidence remains primarily preclinical, and their precise role in glaucoma has yet to be clarified.

As part of this inflammatory response, among the cytokines released by reactive glia, Tumor Necrosis Factor Alpha (TNF-α) has been shown to be one of the central mediators of neuroinflammation and RGC death [21]. In an in vivo rat glaucoma model, TNF-α upregulation occurred within Müller cells and microglia following the induction of OHT, triggering upregulation of AMPA Ca^2+^ receptors on RGCs and causing calcium influx and subsequent RGC death [21].

**Figure 10:**
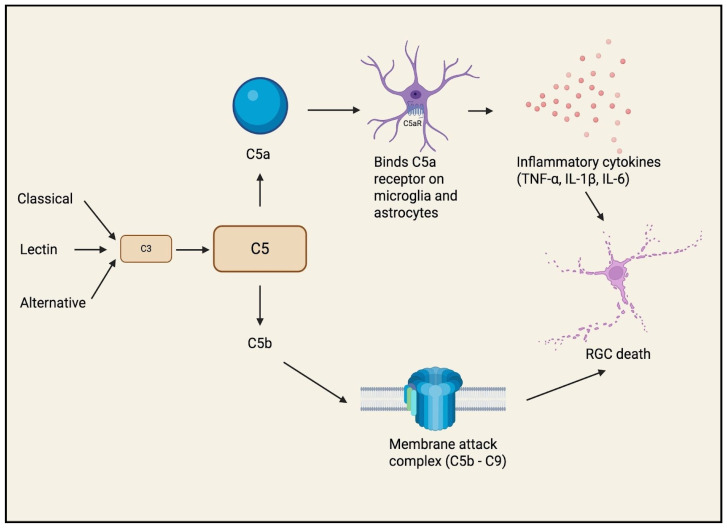
Illustration of the complement cascade, which precipitates retinal ganglion cell death through inflammatory cytokine induction and formation of the membrane attack complex. Created in BioRender. Shome, N. (2026) https://BioRender.com/nt1hqru. **Abbreviations**: TNF-α: tumor necrosis factor α; C: Complement; RGC: retinal ganglion cell.

Another distinct pathway, the complement cascade, has also been shown to be an early and active contributor to RGC death [22]. Components of the classical, lectin, and alternative pathways of the complement system are upregulated in human glaucomatous eyes and animal glaucoma models, preceding overt RGC loss. Activation of the terminal pathway results in the formation of the membrane-attack-complex (MAC: C5b–9) localized in the RGC layer at the ONH, which may injure RGC axons and somas directly (Figure 10) [22]. Beyond these examples, additional mediators such as IL-1β, IL-6, chemokines (e.g., CCL2), toll-like receptor (TLR) signaling, and auto-antibody generation have been implicated in glaucomatous neuroinflammation [3].

Collectively, these findings suggest that neuroinflammation represents a key amplifying mechanism in RGC degeneration, with strong mechanistic support from experimental models demonstrating direct cytokine and complement-mediated neurotoxicity. Notably, complement activation appears to occur early in the disease process, preceding overt RGC loss, raising the possibility that inflammatory pathways may contribute to disease initiation. However, much of the evidence remains derived from animal models and ex vivo human tissue, and the relative contribution of neuroinflammation to disease progression in vivo remains incompletely defined [21,22,23,24]. Taken together, these findings support a model in which elevated IOP not only initiates mechanical and metabolic injury but also contributes to secondary inflammatory and immune amplification of RGC degeneration. This is particularly relevant clinically, as IOP lowering remains the only established modifiable therapy in glaucoma and may therefore help limit both the primary pressure-related insult and at least part of the downstream neuroinflammatory response [1,13,19,20]. Overall, these observations support a model in which glial and immune dysregulation act in concert with oxidative stress and apoptotic signaling to perpetuate RGC injury, while also highlighting neuroinflammation as a promising but not yet clinically validated therapeutic target.

#### 3.3.6. Vascular Dysregulation and Ischemia

As discussed previously, the vascular theory of glaucoma (Figure 5) posits that diminished blood supply may also play a role in the pathogenesis of glaucoma. This is thought to occur because RGCs have high metabolic demand, making them particularly sensitive to local and systemic hypoxia. Several molecular and cellular pathways have been implicated in the ON vascular dysfunction.

One of these pathways involves the molecule endothelin-1 (ET-1), which binds to endothelin receptors (ETR) to induce vasoconstriction. ET-1 levels are known to be elevated within the AH of both human glaucoma patients and animal models of glaucoma [23]. In virally transformed rat RGCs treated with ET-1, increased levels of mitochondrial cytochrome c within the cytosol and increased phosphorylation of c-Jun N-terminal kinase were observed, which led to a higher degree of apoptosis [24]. Similarly, in a comparison between wild-type mice and those with experimentally induced knockout of the ETR gene, researchers found that the wild-type mice demonstrated significantly greater RGC death after 4 weeks of IOP elevation [25].

Nitric Oxide (NO) and cyclic GMP (cGMP) are also believed to play a role in vascular dysregulation [26]. An investigation into the presence of nitric oxide synthase (NOS) in prelaminar and LC astrocytes found that NOS was upregulated in glaucomatous patients with evidence of NO toxicity on histochemical staining of the ON [26]. Another review found that impaired NO-cGMP signaling in the retinal neurovascular unit disrupts neurovascular coupling and vascular regulation, thereby undermining the metabolic support of RGCs and contributing to glaucomatous neurodegeneration [27].

Lesser-known mechanisms involve acid-sensing ion channels (ASICs) and angiotensin II, which are both upregulated in response to low oxygen levels [28]. An evaluation of ASIC1a within rat RGCs in the setting of hypoxia demonstrated upregulation of ASIC1a channels within RGCs, with subsequent increases in intracellular Ca^2+^ influx and RGC cell death as measured by the methyl thiazolyl tetrazolium assay [28]. Similarly, an animal model used to compare the effects of Angiotensin II between an untreated control group and a group treated with losartan, an angiotensin-receptor blocker, found that in the treated group, there were lower levels of α-smooth muscle actin (αSMA), transforming growth factor-β (TGF-β), and collagen type I in the cultured scleral fibroblasts [29].

Collectively, these studies demonstrate that vascular dysregulation in glaucoma extends beyond reduced perfusion to include active neurovascular signaling pathways that directly contribute to RGC injury. Endothelin-mediated vasoconstriction, nitric oxide imbalance, and hypoxia-related ion channel activation have been shown to promote both ischemia and direct neurotoxicity, with evidence spanning human aqueous humor studies and experimental models. However, much of the mechanistic insight is derived from animal and in vitro systems, and the relative contribution of these pathways to disease progression in human glaucoma remains incompletely defined. These findings support a model in which impaired vascular regulation both initiates and amplifies RGC degeneration, while highlighting the need for further clinical validation of vascular-targeted therapies.

#### 3.3.7. Apoptotic Pathway

**Figure 11:**
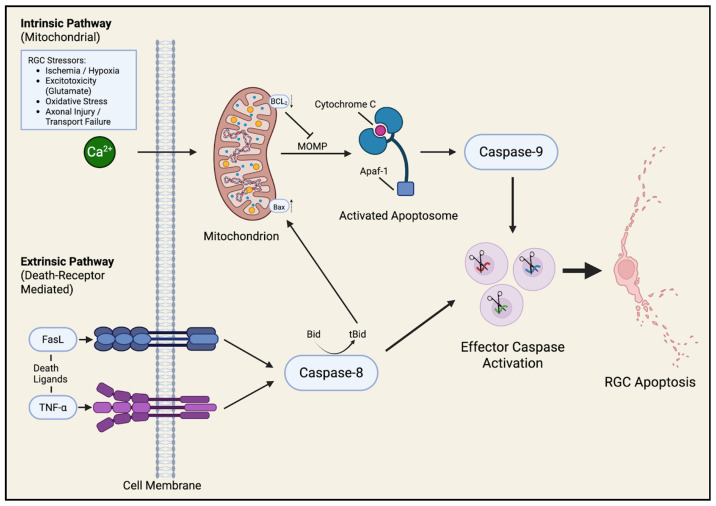
Diagram of the intrinsic (mitochondrial) and extrinsic (death-receptor mediated) apoptotic pathways. Both pathways converge upon effector caspase activation and retinal ganglion cell apoptosis. Created in BioRender. Shome, N. (2026) https://BioRender.com/3nrsxgp. **Abbreviations**: RGC: retinal ganglion cell; FasL: Fas ligand; TNF-α: tumor necrosis factor α; MOMP: mitochondrial outer membrane permeabilization.

Apoptosis represents the final common pathway through which multiple glaucomatous stressors (mechanical strain, oxidative and mitochondrial dysfunction, trophic factor deprivation, vascular compromise, and neuroinflammation) culminate in irreversible loss of RGCs [13]. Apoptosis, or programmed cell death, ensures controlled elimination of irreparably damaged neurons, yet in glaucoma this process becomes pathologically activated, driving progressive neurodegeneration even after the primary insult has subsided. Two major apoptotic cascades, the intrinsic (mitochondrial) and extrinsic (death-receptor mediated) pathways, have been most clearly implicated in glaucomatous optic neuropathy (Figure 11).

The intrinsic or mitochondrial pathway is triggered by intracellular stress signals such as oxidative damage, calcium overload, DNA injury, or loss of neurotrophic support [30]. These stimuli disrupt the balance of pro and anti-apoptotic members of the Bcl-2 family, tipping the scale toward activation of Bax, Bak, and related effectors that permeabilize the outer mitochondrial membrane. This event, known as mitochondrial outer membrane permeabilization (MOMP), results in the release of cytochrome c, Smac/DIABLO, and other apoptogenic factors into the cytosol. Cytochrome c binds to Apoptotic protease activating factor 1 (Apaf-1) to form the apoptosome, which activates caspase-9 and subsequently caspase-3, executing cell death through DNA fragmentation and cytoskeletal breakdown [30]. Experimental glaucoma models consistently show up-regulation of Bax and cleaved caspase-3, together with down-regulation of Bcl-2, supporting a mitochondria-driven mode of RGC loss [31]. Mitochondrial fragmentation, impaired ATP synthesis, and oxidative stress further reinforce this pathway, creating a self-amplifying cycle of energy failure and apoptosis.

The extrinsic pathway, in contrast, is initiated by ligand binding to specific death receptors on the RGC membrane [32]. Glial cells within the glaucomatous retina and ON release death cytokines such as TNF-α and express Fas ligand (FasL), which interact with their respective receptors TNFR1 and Fas on neighboring neurons. Ligand engagement recruits adaptor proteins like FADD and procaspase-8 to form the death-inducing signaling complex (DISC), leading to activation of caspase-8 and downstream effector caspases. Activated caspase-8 can also cleave Bid into tBid, which translocates to mitochondria to promote MOMP, thus bridging the extrinsic and intrinsic cascades [32]. Through this mechanism, inflammation-driven signaling by reactive astrocytes and microglia can directly translate into apoptotic execution in adjacent RGCs.

Beyond classical apoptosis, several alternative forms of regulated cell death have been described in experimental glaucoma. The molecular mediators and the role of mechanisms in RGC dysfunction are shown in more detail in Table 3. Although these non-canonical pathways remain under investigation, they likely modulate or complement classical apoptotic signaling rather than replace it as the principal mechanism of RGC loss.

Collectively, these studies also establish apoptosis as the primary and most consistently supported mechanism of RGC death in glaucoma, with converging evidence from multiple experimental models demonstrating activation of both intrinsic mitochondrial and extrinsic death receptor pathways. However, emerging data suggest that apoptosis does not occur in isolation, as parallel processes such as ferroptosis and autophagy-related cell death may contribute to or modulate this degenerative cascade. Importantly, the majority of evidence is supported and derived from animal and in vitro systems, and the extent to which these pathways drive RGC loss in human glaucoma is incompletely understood. These findings uphold apoptosis as a central but not exclusive mechanism of neurodegeneration, highlighting the need for therapeutic strategies that account for overlapping cell death pathways.

#### 3.3.8. Summary of RGC Pathophysiologic Mechanisms

Taken together, these mechanisms do not act in isolation but rather form an interconnected network of injury pathways. Mechanical stress, vascular compromise, and metabolic dysfunction converge to disrupt axonal transport, induce oxidative stress, and activate inflammatory cascades, ultimately culminating in apoptotic RGC death. This integrated model highlights the limitations of single-target therapies and supports the need for multimodal approaches to neuroprotection in glaucoma.

### 3.4. Shared Mechanisms of Degeneration in Common Optic Neuropathies

Similar to GON, other optic neuropathies share some common neural damage pathways, such as ischemia [nonarteritic anterior ischemic optic neuropathy (NAION) and arteritic anterior ischemic optic neuropathy (AAION)], inflammation/demyelination [optic neuritis, neuromyelitis optica spectrum disorder (NMOSD), myelin-oligodendrocyte-glycoprotein antibody–associated disease (MOGAD)], mitochondrial [leber hereditary optic neuropathy (LHON) and dominant optic atrophy (DOA)] and compressive optic neuropathies (CON). All optic neuropathies share three underlying common mechanisms of damage to RGCs: axonal transport disruption [36], mitochondrial dysfunction [37], and inflammatory insults [38].

Although many optic neuropathies share overlapping mechanisms of retinal ganglion cell death with glaucoma, important differences exist in the pattern of retinal ganglion cell loss, underlying pathology, and associated clinical findings. Table 4 summarizes these differences by comparing characteristic OCT findings, pathogenic mechanisms, and representative visual features across glaucoma and selected optic neuropathies.

### 3.5. Progression Monitoring of RGC Changes in Glaucoma and Early Detection

#### 3.5.1. How to Monitor RGC Changes in Glaucoma: A Combination of Structural and Functional Measures and Integration of Multiple Biomarkers

##### Structural Biomarkers of RGC Damage

**Figure 12:**
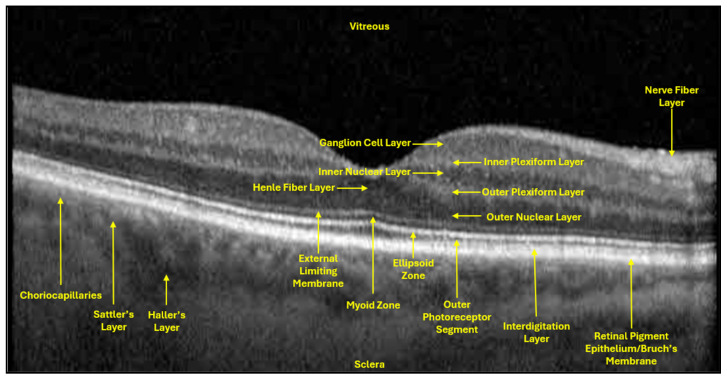
B-scan of the retina showing the various layers of retina and choroid.

In clinical ophthalmology, structural imaging and diagnostics remain the backbone for monitoring glaucomatous neurodegeneration. Peripapillary RNFL thickness is measured using OCT, and it is the most widely validated structural biomarker for axonal loss. OCT functions to generate horizontal and vertical two-dimensional cross-sectional images by measuring the echo-time delay of reflected light (measures depth) and the optical backscatter (measures intensity of reflected light to generate image brightness) (Figure 12). RNFL exists as the chief structural biomarker of RGC damage in glaucoma due to the lack of direct visualization of RGCs [50].

The GCL and IPL are additional structural biomarkers that can be used in the diagnosis of RGC damage in glaucoma (Figure 12). The RNFL contains RGC axons and GCL somata, while the adjacent IPL, where RGC dendrites synapse with bipolar and amacrine cells, is the primary site of excitatory–inhibitory integration and an early site of glaucomatous damage [51]. El-Danaf and Huberman used fluorescent labeling of RGC subtype proteins to map dendritic rearrangements, showing early remodeling within the OFF sublamina of the IPL during the first week of elevated IOP. This remodeling involved reduced dendritic length and density in the OFF layer, while ON sublamina dendrites remained largely unaffected [52,53]. This study suggests that further exploration of spatially distinct RGC dendritic rearrangements may serve as a promising structural biomarker in early glaucoma before irreversible somatic damage.

In addition to RNFL, both GCL and IPL thickness are also measured using OCT. GCL and IPL thinning are both strongly correlated with glaucoma disease progression. In a 2018 prospective cohort study, 93 patients were stratified by stage of glaucoma (mild, moderate, severe), and GC-IPL thickness declined significantly with disease progression [54]. Moreover, in that same study, structure-function relationships were also well documented, as a 1 µm decrement in average GC-IPL thickness was associated with a ~0.54 dB loss in VF MD [54].

##### Functional Biomarkers of RGCs

**Figure 13:**
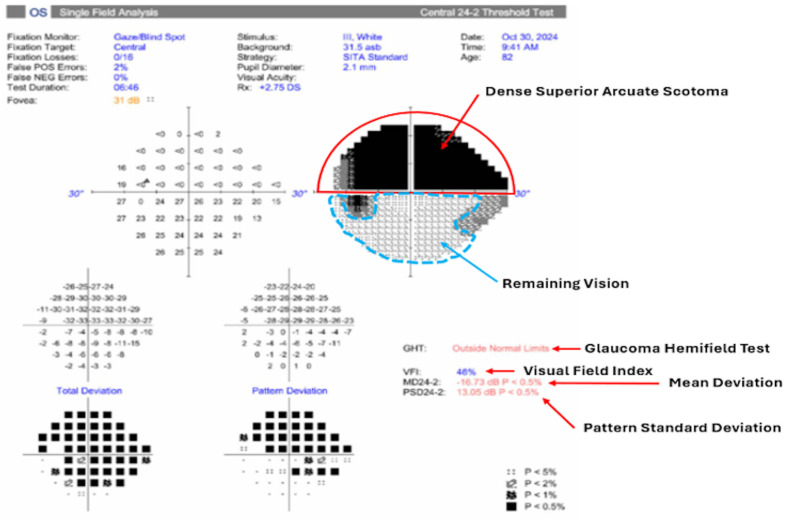
Visual field results of a right eye with advanced glaucoma showing a superior arcuate scotoma. Glaucoma hemifield test = outside normal limits, visual field index = 46%, mean deviation = −16.73 dB, pattern standard deviation = 13.05 dB are listed.

VF deterioration, measured with standard automated perimetry (SAP) VF, can be strongly correlated with progressive thinning of the superior and inferior RNFL sectors (Figure 13). VF tests only detect defects once a substantial proportion of RGCs (~25–35%) have already been lost, meaning that perimetry may not be helpful in early stages of the disease cascade [55]. Contrarily, current clinical tools like OCT and VF only detect damage after significant RGC loss (20–40% reduction of RGCs), corresponding to a diagnostic delay of up to 10 years. However, when used clinically, the subjective nature of VF (reliable fixation, patient responses, fatigue, learning effects) can oftentimes impose limitations on accurate reproducibility [56].

**Figure 14:**
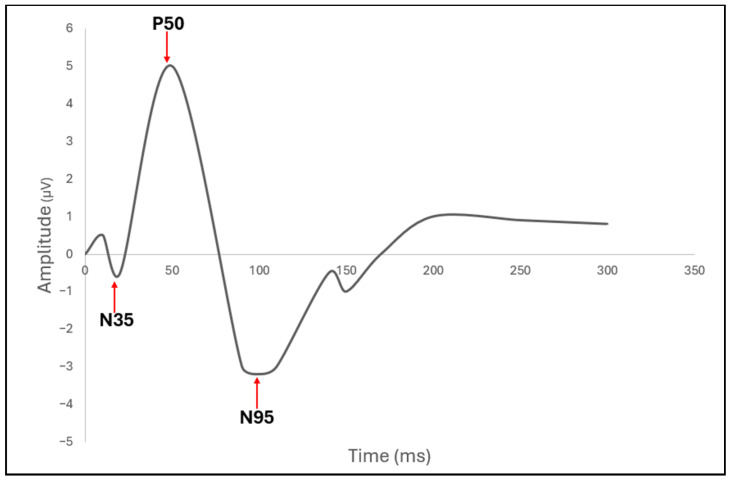
Sample pattern electroretinogram test wave showing the N35 (small negative wave at ~35 ms) = early retinal activity, P50 (large positive wave at ~50 ms) = outer retinal/cone function, and N95 (large negative wave at ~95 ms) = retinal ganglion cell function.

**Figure 15:**
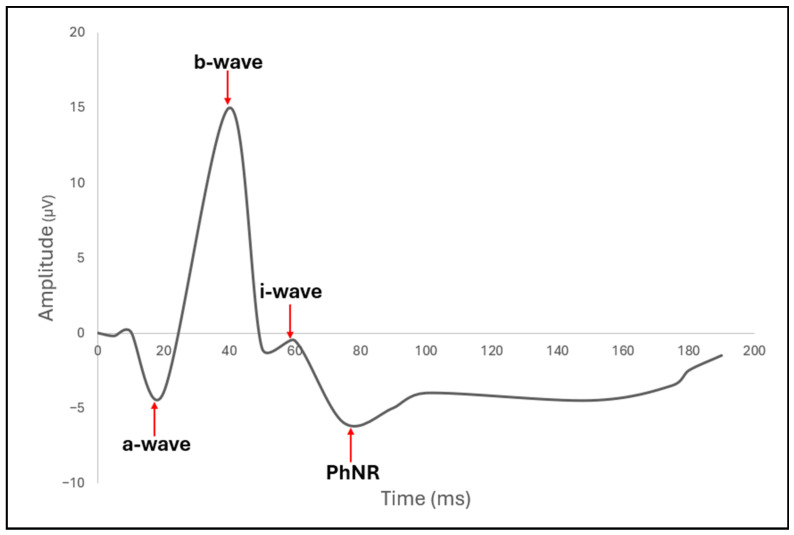
A sample of photopic negative response test wave showing the a-wave (first negative trough) = photoreceptor function, the b-wave (first positive peak after the a-wave) = bipolar cell activity, the i-wave (transient positive deflection around 60 ms) = amacrine/bipolar cell interaction, and the photopic negative response (a slow negative wave after the b-wave) = retinal ganglion cell function.

To overcome some of the sensitivity and subjectivity limitations of VF, electrophysiological measures have emerged as powerful functional biomarkers of RGC integrity. Two of the most established are the pattern electroretinogram (PERG) (Figure 14) and the photopic negative response (PhNR) (Figure 15) of the full-field electroretinogram (ERG). A 2013 study compared PhNR and PERG across glaucoma stages and showed that both were reduced in early (preperimetric) and manifest glaucoma [57]. Additionally, the study also discovered the ratio metrics (e.g., PhNR to b-wave, PERG 0.8°/16°) often outperformed raw amplitudes in discriminating disease, linking both to increased detection of glaucoma [57]. The clinical applications of PERG and PhNR are further discussed later in this section.

**Figure 16:**
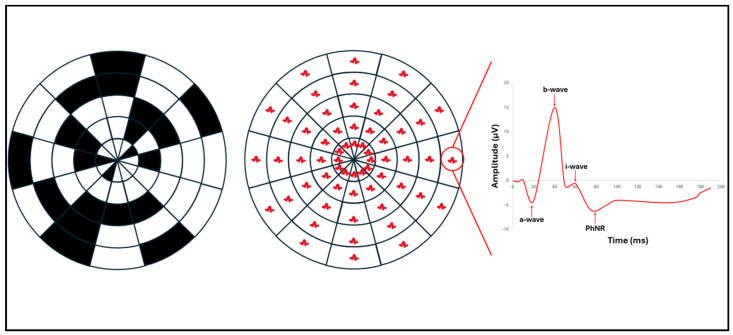
Schematic of multifocal PhNR (mfPhNR) recording. Localized visual stimuli are presented across the visual field to generate focal ERG responses, from which the photopic negative response (PhNR) is extracted as the negative amplitude following the b-wave, showing regional retinal ganglion cell function. Adapted from [58]. **Abbreviations**: PhNR: phototopic negative response.

One limitation of full-field PhNR (ffPhNR) is its lack of spatial specificity for detecting localized RGC dysfunction. Multifocal PhNR (mfPhNR) addresses this by providing topographic mapping of RGC function, enabling more sensitive detection and longitudinal tracking of regional glaucomatous damage (Figure 16) [58]. Complementing this, the mesopic negative response (MeNR) probes rod-driven RGC pathways under mesopic conditions, offering an additional functional biomarker that may capture aspects of RGC dysfunction not assessed by conventional PhNR testing [58,59].

##### Cellular Biomarkers

Cellular biomarkers of RGC damage in glaucoma can indicate the neurodegenerative processes occurring at the level of the soma, axon, mitochondria, and supporting glial cell networks. The goal of reaching clinical neuroprotective therapies of the RGCs first requires tools to accurately quantify RGC survival. In human patients, RGC loss cannot be counted in vivo, as this requires post-mortem retrograde tracing and immunohistochemical staining [60]; thus, clinicians instead rely on surrogate cellular estimates derived from high-resolution imaging (OCTA and VF). Although apoptosis, dendritic pruning, and axonal degeneration cannot be visualized directly, their aggregate structural/functional signatures (regional thinning, localized axonal dropout, and ONH deformation) are clinically measurable and serve as reliable reflections of cellular pathology. Contrarily, in vivo animal models allow researchers to directly quantify RGC damage throughout the progression of glaucoma, which is used to help validate the cellular processes that most strongly correlate with clinically observed structural degradation [61,62].

Among clinically relevant cellular biomarkers in RGC damage in glaucoma, apoptosis is the most significant. The results of apoptosis include loss of tissue volume and progressive thinning of the GCL. Patterns identified in human imaging, such as focal thinning in the macular vulnerability zone, strongly correlate with these apoptotic topographies, indicating that apoptosis is a biologically essential, albeit clinically inferred, cellular biomarker. Additionally, researchers are currently exploring a new technology called detection of apoptosing retinal cells (DARC) to noninvasively capture real-time visualization of apoptotic changes of RGCs [63] (for details, see Section 3.6.2).

Another major cellular biomarker is RGC axonal degeneration, specifically within the ONH. In humans, although axons cannot be individually imaged, their degeneration may appear as neuroretinal rim thinning, progressive cupping, and localized RNFL defects on OCT. These symptoms serve as clinically relevant representations for the essential cellular process of axonopathy. Longitudinal clinical studies have shown that these axonal biomarkers strongly correlate with functional decline, which highlights their relevance to disease monitoring [64].

Mitochondrial dysfunction is another important cellular biomarker of RGC stress. One unique structure of RGCs is that their axons are unmyelinated in the RNFL but become heavily myelinated after they pass the LC. Therefore, RGCs require high ATP output due to their long unmyelinated intraocular retinal axons, rendering them vulnerable to mitochondrial injury. Experimental mouse models in a 2008 study demonstrated mitochondrial matrix swelling, oxidative stress, and decreased respiratory chain function early in glaucoma, often preceding structural degeneration [65]. In humans, these mitochondrial deficits cannot be imaged directly, but their downstream effects are reflected in region-specific VF vulnerability patterns and functional abnormalities on electrophysiologic testing (PERG & PhNR) [66].

A further clinically relevant biomarker is glial cell activation, specifically those involving microglia and Müller cells (retinal macroglia). In human patients, this activation is inferred clinically from subtle OCT changes, such as hyperreflective foci, alterations in RNFL texture, or accelerated thinning in regions of inflammatory stress [67]. Although these imaging correlates are indirect, they are capable of reliably tracking the retinal areas where glial-mediated secondary injury is active.

Finally, alterations of the RGC synapses within the IPL represent another early cellular biomarker of RGC stress in glaucoma. Experimental studies indicate that dendritic retraction, synaptic loss, and LC remodeling often occur before soma loss becomes detectable [68,69]. In clinical ophthalmology practice, these structural changes to the RGC synapses are identified through subtle OCT reflectivity changes, sectoral VF vulnerability patterns, and early functional impairments (such as reduced PERG amplitudes) in regions without major thinning. Therefore, synaptic integrity represents a sensitive early indicator of RGC dysfunction, but its clinical use remains limited by the lack of direct, standardized in vivo measurement techniques.

##### Integrating Structural, Functional, and Cellular Biomarkers

Glaucoma is widely recognized as a multifactorial neurodegenerative disease in which structural loss, functional deficiency, and cellular injury represent the interconnected stages of a chronic progressive disease rather than untimely isolated events.

Functional biomarkers complement structural measures by capturing the physiologic consequences of RGC dysfunction, often preceding irreversible tissue loss. Electrophysiological tests (PERG, PhNR, mfPhNR) detect early alterations in RGC signaling, while SAP-VF reflects the downstream impact on visual perception. It is essential to recognize that functional deficits in regions without apparent thinning can often be attributed to early cellular or synaptic dysfunction. This highlights the value of functional testing in detecting potentially early, reversible stages of glaucoma.

Combining structural, functional, and cellular biomarkers enables a more comprehensive assessment of RGC damage in glaucoma. Structural imaging estimates the physical effects of RGC loss, functional testing reveals ongoing visual impairment, and cellular biomarkers provide context for both. Understanding these biomarkers should help improve early detection, enhance longitudinal monitoring of progression, and support a shift toward personalized glaucoma care. In current practices, these decisions should not be guided by one or two metrics, but instead use a multimodal approach, combining clinical, retinal imaging, and functional VF loss as evidence of RGC health.

#### 3.5.2. Detecting Changes over Time

##### Earliest Detectable Changes

The earliest detectable changes predisposing to glaucoma often occur at the TM level and include (1) iridotrabecular contact in angle closure, (2) increased TM pigmentation from pigment dispersion syndrome, and (3) increased lens thickness causing anterior chamber crowding. These anatomical changes can be identified through gonioscopy and anterior segment imaging before functional damage occurs [70].

Before RNFL cell loss and VF defects, glaucoma has been shown to demonstrate functional stress in RGCs. Many of the earliest detectable changes in glaucoma can be better identified with PERG and PhNR than OCT-RNFL, as these electrophysiologic tests can accurately track RGC defects and predict the future severity of glaucoma [71,72]. PERG captures three wavelength amplitudes, N35, P50, and N95, which represent early retinal function, outer retinal macular cones, and RGCs, respectively (Figure 14 and Figure 15). Clinicians utilize high-frequency steady-state PERG (reversal frequency of 8 Hz or 16 reversals per second) to help decrease gaze fixation loss in early steady-state (slower reversal rate) PERG. In response to early RGC damage in glaucoma, PERG exhibits decreased N95 amplitudes [73].

Because PhNR is elicited by a flash stimulus (typically red on a blue background), it is less dependent on optics, refractive correction, or stable fixation, giving it an advantage in populations where perimetry or PERG may be difficult. In response to early RGC damage in glaucoma, PhNR exhibits reduced b-wave amplitudes. Although PERG and PhNR provide unique advantages in the diagnosis of RGC damage in glaucoma, these tests are often less clinically widespread due to their lack of large-scale clinical robustness, standardization, and specificity.

Though PERG and PhNR can be utilized to identify RGC defects early in glaucoma, OCT-RNFL/GCIPL and VF SAP are still the preferred diagnostic tests for clinical ophthalmologists [74]. Using clinical VF and RNFL data, Harwerth et al. proposed an empirical formula to quantify estimated RGC counts from factors such as retinal eccentricity, axonal density, VF sensitivity, and VF-specific VA [75]. This experimental formula, which was based on experimental studies of rhesus monkeys and translated to human clinical SAP data, has been validated in glaucoma and offers a more accurate diagnosis of early-stage glaucoma and a better prediction of disease progression [76]. One study indicated that at the early stage of glaucoma, significant decreases in estimated RGCs were strongly correlated with relatively small SAP mean deviations (MD) [77]. This finding corroborates the current data that suggests ONH/RNFL degenerates before statistically significant SAP defects are observed in the VF [75].

##### Moderate and Advanced Glaucoma Changes

In moderate to advanced glaucoma, measurable thinning of the RNFL/GCC is observed across large retinal sectors, causing pronounced ONH cupping. Additionally, as glaucoma progresses, RNFL thickness may ultimately plateau due to the “floor effect”, or the point at which the RNFL becomes so thin (approximately 30–45 µm) that further structural damage cannot be detected by the OCT device [78]. In relation to RGCs, this progressive advancement causes the commonly found arcuate scotoma measured by VF testing (Figure 13). In severe cases of glaucoma, MD values exceed −12 dB, with some patients reaching −20 dB or worse. Patients with severe glaucoma often have central field involvement (macula), where approximately 50% of all RGCs are located. Some of the values used to determine the severity of glaucomatous damage on a VF report are: glaucoma hemifield test (indication of inter-hemifield visual differences), visual field index (visual field as a percentage of normal for that age group), mean deviation (average deviation of the entire visual field compared to age-matched norms), and pattern standard deviation (threshold difference between the depressed areas versus the healthy surrounding areas) (Figure 13).

RGC estimation can also be utilized in moderate to advanced glaucoma. Based on the formula used for early detectable glaucoma changes first proposed by Harwerth et al. [75], Medeiros et al. developed a new formula incorporating weighted integration of density (age-related decline in axonal density) and severity correction (adjusts for decreasing axonal to non-axonal ratio in RNFL) that accounted for more variable macular damage in severe glaucoma [79]. In a clinical study by Wu et al., analysis of 119 eyes across preperimetric to advanced glaucoma stages showed that reductions in estimated total RGCs were consistently proportional to significant declines in macular RGC estimates. This model suggests that the high-density mRGCs are promising sensitive indicators in both the diagnosis and follow-up of progressive glaucoma [76].

Finally, though PERG and PhNR may be useful in identifying early RGC loss in glaucoma, little research exists to suggest significant changes in wavelength amplitudes between the progressing stages of glaucoma due to the high inter-individual variability of these tests. Rather than tracking RGC damage during the progression of glaucoma, these tests may be helpful in identifying early RGC damage before VF or RNFL defects are evident.

#### 3.5.3. Factors Influencing Progression Speed

The tempo at which RGCs degenerate in glaucoma is determined by the interaction of both the magnitude and duration of the primary stressor. Many additional factors that may influence progression speed include age, race, genetic inheritance, gender, corneal thickness, and myopia. Though not controllable, these factors may contribute to RGC damage with or without IOP elevation. Patients with these risk factors may require varying levels of screening and therapeutic intervention in relation to IOP control.

Predicting the progression of RGC damage, therefore, requires integrating mechanical, vascular, metabolic, cellular, inflammatory, and clinical modifiers into a longitudinal framework (see Section 3.3). Of the mechanisms that are more fully understood, elevated IOP (mechanical theory of glaucoma) is the single most significant modifiable risk factor for maintaining RGC function. Peak IOP, pattern of IOP elevation (chronic low-grade vs. acute spikes), and the duration of IOP elevation are the primary determinants of how quickly RGC injury advances. In a rat model study, researchers induced a 100% increase in IOP, which subsequently showed a statistically significant decline in ERG amplitude after 3 months, while mitochondrial membrane potentials decreased by 17.5%. After 4 months, apoptosis and functional loss from stress were observed. Therefore, achieving lower IOP is the principal goal to maintain RGC function in glaucoma, as a 20% decrease in IOP has been shown to slow the disease progression by 50% [80].

#### 3.5.4. Reversible vs. Irreversible Changes

##### Reversible Changes

It was originally believed that all forms of damage to RGCs were irreversible by nature. However, recent clinical evidence suggests that reversible changes to RGCs can occur and typically precede the more severe irreversible changes, such as apoptosis and Wallerian degeneration. The first and most prominent reversible change in RGCs is dysfunction. In the early stages of glaucoma, RGC stressors induce neuroprotective strategies and glial cell activation to re-establish homeostasis and enter a “comatose” state. Mechanisms of dysfunction work to decrease metabolic demand and buy time for cellular recovery if the initial stressors of glaucoma are removed. These mechanisms include altering ion channel function/membrane conductances and retracting synaptic contacts in the IPL, thereby decreasing neuronal excitability, neurotransmitter release, and ATP consumption [81]. A 2016 study showed that an acute increase in IOP in mice resulted in progressive thinning of the IPL over a month, demonstrating possible synaptic and dendritic damage [82]. This highlights the clinical importance of detecting early IPL alterations as a potentially reversible stage of RGC injury and an early window for therapeutic intervention.

##### Irreversible Changes

The most common form of irreversible RGC damage originates from regulated cell death, commonly referred to as apoptosis (see Section 3.3.7). This process may be reversed if the inciting stress is adequately alleviated. However, persistent injury to the RGCs triggers active cellular death and DNA fragmentation. The rapid disease progression may lead to functional deficits that are diagnosed and monitored using VF and OCT.

Another form of RGC irreversible damage in glaucoma is Wallerian degeneration of the axon [83]. Also known as anterograde/orthograde degeneration, this process occurs when both the cytoskeleton and myelin of the RGC axon disintegrate in the part of the nerve that is distal to the site of injury (away from the cell body). These events normally occur within the first 24 h of meeting the Wallerian degeneration threshold, with severe nerve damage occurring within a week [84]. A 2012 study by Wang et al. found that, in mice, there was significantly delayed damage in those with the mutant Wallerian degeneration slow (Wld^s^) gene compared to those with the wild type gene [83]. In this study, the subject eyes with Wld^s^ exhibited delayed functional loss at one week, fewer total RGC losses after two weeks, and decreased morphological ONH observations overall [85]. These findings demonstrate that modulation of the Wallerian degeneration pathway can potentially delay axonal and somatic RGC loss, which highlights a possible target for neuroprotection.

#### 3.5.5. Frequency of Assessment Recommendations

The American Academy of Ophthalmology recommends a baseline comprehensive eye exam by age 40, with follow-up intervals ranging from every 2–4 years (ages 40–54), every 1–3 years (55–64), and every 1–2 years (≥65) in individuals without risk factors [86]. In practice, surveillance frequency is individualized based on risk profile, disease stage, and clinical objectives, with evidence from a multicenter trial suggesting that biannual assessments are effective for detecting rapidly progressing glaucoma. However, intervals should be shortened if progression occurs despite treatment or the target IOP is not achieved [87,88].

To reduce the risk of irreversible vision loss due to glaucoma, clinical ophthalmologists are striving to improve the early detection of glaucoma through screening programs. In addition, in patients with cataracts, the recommendation is to combine cataract surgery with minimally invasive glaucoma surgery (MIGS) in appropriate candidates. By lowering IOP earlier in the disease course—before significant structural damage occurs—and reducing medication burden, combined cataract/MIGS procedures may interrupt the cascade of events leading to irreversible RGC death. Follow-up studies concerning MIGS, such as the HORIZON randomized trial, demonstrate greater probabilities of VF preservation, supporting the concept that earlier intervention protects against progressive RGC loss [89].

### 3.6. Advanced Imaging and AI in RGC Assessment

#### 3.6.1. Challenges and Pitfalls in RGC Imaging

Direct visualization of RGCs remains an unresolved challenge in clinical imaging due to a combination of biological and optical limitations. RGCs are small, low-contrast, and located within the inner retina, where their reflectivity is overshadowed by adjacent structures such as nerve fiber bundles and blood vessels [90]. As a result, conventional SD-OCT (discussed in Section 3.5) does not have the resolution or signal discrimination needed to identify individual RGC somas, forcing clinicians to rely on surrogate markers such as GCC and RNFL [90].

Advanced imaging modalities such as adaptive optics scanning laser ophthalmoscopy (AO-SLO) and adaptive optics OCT offer the potential to resolve cellular-level detail, but these techniques remain technically complex and highly sensitive to optical aberrations, motion artifacts, and scattering from inner retinal structures [90]. Their limited field of view, long acquisition times, and alignment requirements limit their utility in routine clinical practice. Additionally, biological heterogeneity of RGC subtypes and variability in RGC soma size, dendritic arbor geometry, and relative positioning within the ganglion cell layer make it difficult to standardize cell-level imaging metrics [90]. Together, these limitations highlight why direct RGC visualization is still not feasible in clinical glaucoma management, instead requiring measurements of inner retinal layers.

#### 3.6.2. Emerging High-Resolution Imaging and Advanced Technologies

Recent advances in retinal imaging have enabled increasingly detailed visualization of structural and microvascular changes relevant to RGC degeneration (Table 5). Ultrahigh-resolution OCT (UHR-OCT), with axial resolution approaching 2–3 µm, can reveal microstructural alterations within the inner and outer retina that are not detectable using conventional SD-OCT [91].

Adaptive optics–based imaging, including AO-OCT and AO-SLO, achieves cellular-level lateral resolution by correcting ocular aberrations. Although technically demanding and currently limited to research settings, these modalities demonstrate the potential for direct visualization of RGC somas and dendritic architecture [90].

**Figure 17:**
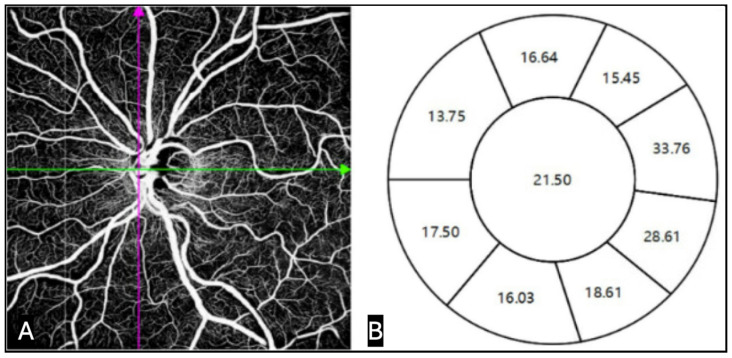
(**A**). Swept-source optical coherence tomography angiography image of the optic nerve and radial peripapillary capillaries. (**B**). Corresponding vessel densities.

Swept-source OCT angiography (SS-OCTA) has emerged as a clinically feasible tool for assessing microvascular networks supplying RGC axons (Figure 17). Studies consistently show that vessel density loss measured by SS-OCTA correlates strongly with RNFL and GCC thinning and may precede functional decline, positioning SS-OCTA as a promising modality for early detection and longitudinal monitoring of glaucomatous neurovascular compromise [92]. While these findings suggest a potential role for OCTA in early detection and monitoring, variability in acquisition, segmentation, and interpretation continues to limit standardization. This can be compounded by artifacts, projection errors, and limited sensitivity to slow changes in microvasculature, decreasing reliability for routine clinical decision-making.

**Figure 18:**
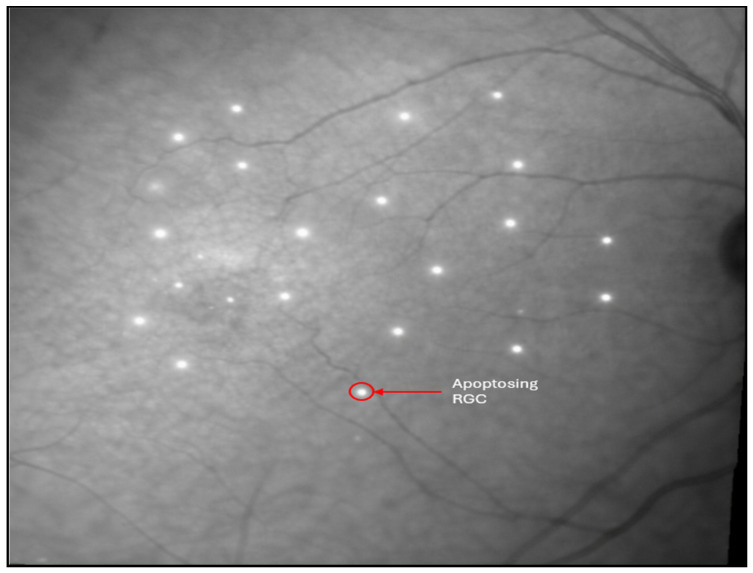
Fundus image showing scattered punctate hyperfluorescent signals representing actively apoptosing RGC in glaucoma using DARC. Original creation by the authors. **Abbreviations**: RGC: retinal ganglion cell.

Detection of apoptosing retinal cells (DARC) is a promising technology that is able to detect RGC apoptosis in vivo before irreversible VA loss. DARC enables real-time visualization of apoptosing RGCs by using fluorescently labeled Annexin V to bind phosphatidylserine on stressed cells, which are then imaged and quantified as discrete signals using confocal scanning laser ophthalmoscopy (cSLO) (Figure 18). Preclinical studies demonstrated the feasibility of detecting single RGC apoptosis events in vivo, and early translational work showed that DARC counts correlate with disease activity and IOP-related injury rather than late structural loss alone [93]. In a series of studies, Cordeiro et al. demonstrated continuous imaging of RGC apoptosis in animal models [93]. This highlights DARC as a potentially promising direct cellular biomarker of neurodegeneration rather than an indirect estimate of structural or functional measure, but more robust clinical validation is needed before DARC can be implemented for use in clinical glaucoma diagnosis.

**Table 5:** Summary of emerging imaging technologies for RGC assessment.

Technology	Key Principle	Strengths	Limitations
UHR-OCT [90]	Ultra-high axial resolution retinal imaging using broadband light sources	Detects microstructural changes associated with RGC degeneration	Limited accessibility, high cost
AO [90]	Correction of ocular aberrations to enable cellular-level retinal imaging	Potential for direct RGC visualization	Small FOV, motion artifacts
SS-OCTA [91]	High-speed depth-resolved retinal imaging using a swept tunable laser source	Captures microvascular dropout of peripapillary vessels (which supply RGC axons)	Motion artifacts, variable scan quality, GCC assessment dependent upon segmentation algorithms
DARC [93]	In vivo imaging of apoptosing RGCs using fluorescently labeled annexin V	Can identify RGC death before irreversible vision loss	Invasive (requires intravitreal injections), limited clinical validation, not glaucoma specific

**Abbreviations**: UHR-OCT: Ultrahigh resolution optical coherence tomography; FOV: Field of view; AO: Adaptive optics; SS-OCTA: Swept source optical coherence tomography angiography; RGC: retinal ganglion cell; GCC: ganglion cell complex.

#### 3.6.3. Machine Learning for Structural Pattern Recognition

Artificial intelligence (AI) approaches have increasingly been applied to structural imaging data to detect glaucomatous damage in RGC-related layers. Machine learning (ML) and deep learning (DL) models trained on OCT or OCTA-derived features can distinguish glaucomatous from healthy eyes based on complex patterns in RNFL, GCIPL, and vessel density maps that are difficult to appreciate visually [94]. For example, convolutional neural networks (CNNs) trained on en face OCTA images of the radial peripapillary capillary network have achieved high accuracy for classifying glaucoma versus controls, outperforming traditional vessel density summary metrics and gradient-boosting classifiers [94]. Similarly, geometric DL and explainable AI frameworks applied to single OCT scans of the ONH and peripapillary retina have shown strong performance for glaucoma diagnosis and staging, indicating that subtle alterations in RGC axons and their support tissues contain useful diagnostic information [94,95]. However, most current evidence remains cross-sectional and model-dependent, with limited external validation and standardized interpretability. Therefore, AI frameworks do indicate promise for diagnostic support, but significant longitudinal validation is needed prior to widespread clinical use.

#### 3.6.4. Deep Learning for Early Glaucoma and Progression Prediction

Beyond static classification, DL models have been developed to link RGC-related structural measurements directly to functional loss and future disease trajectory. Several groups have trained CNNs on macular GCIPL and peripapillary RNFL thickness maps to predict SAP outcomes, including global VF indices and pointwise sensitivity, demonstrating that OCT-derived surrogates of RGC structure can accurately estimate the severity and spatial pattern of VF damage [96,97]. More recently, longitudinal DL systems using serial OCT scans have been used to forecast future RNFL thinning in glaucoma cohorts, suggesting a role for AI in predicting progression risk based on trends in RGC-related structural loss rather than relying solely on retrospective change analysis [98,99].

Additionally, emerging AI-based tools and validated risk calculators may offer promising approaches for community-based glaucoma screening. The Laroche glaucoma calculator, which integrates IOP, CCT, and age, has demonstrated 93.5% sensitivity and 91.3% specificity in high-risk populations, enabling trained non-physician personnel to identify individuals requiring comprehensive evaluation [100]. Such tools, combined with large registry data (e.g., IRIS Registry), may facilitate earlier identification of glaucoma suspects and enable timely intervention. Despite these promising results, current AI models face important limitations, including dependence on device-specific imaging protocols, restricted training populations, and a lack of transparent biologic interpretability at the level of individual RGC subtypes [101]. Most systems are trained on established glaucoma rather than truly preperimetric disease, and few have been prospectively validated or integrated into clinical workflows. As larger, multimodal datasets (combining OCT, OCTA, and clinical risk factors) become available, AI has the potential to complement traditional structural metrics by refining early detection, individualizing progression risk estimates, and more directly capturing the health of RGC-related retinal circuitry.

### 3.7. Future Therapeutics

#### 3.7.1. Excitotoxicity, Mitochondrial Dysfunction, and Oxidative Stress

**Figure 19:**
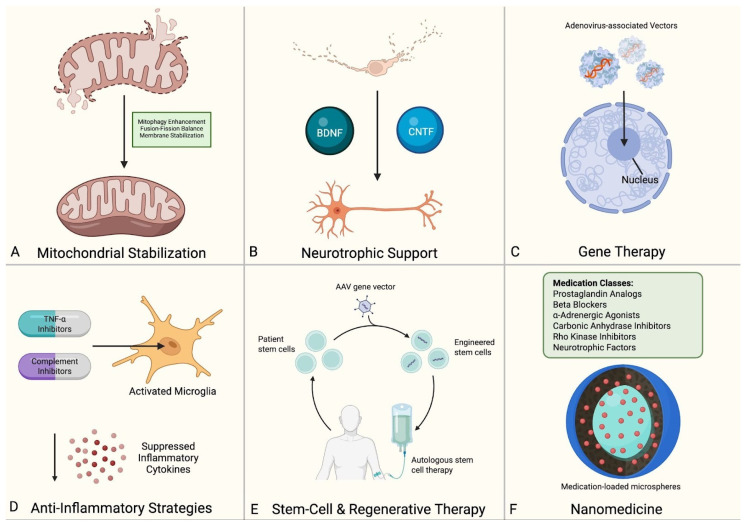
Summary of future therapeutic approaches for retinal ganglion cell neuroprotection. (**A**) Mitochondrial stabilization. (**B**) Neurotrophic support. (**C**) Gene therapy. (**D**) Anti-inflammatory strategies. (**E**) Stem cell and regenerative therapy. (**F**) Nanomedicine. Created in BioRender. Shome, N. (2026) https://BioRender.com/b4cmf2d. **Abbreviations**: BDNF: brain-derived neurotrophic factor; CNTF: ciliary neurotrophic factor; TNF-α: tumor necrosis factor α. AAV: adeno-associated virus.

While current glaucoma management focuses primarily on IOP reduction, the multifactorial pathophysiology of RGC death presents numerous alternative therapeutic targets. The following section examines emerging therapies organized by their primary mechanism of action, such as excitotoxicity, oxidative stress, mitochondrial dysfunction, neurotrophic factor deficiency, as well as potentially transformative approaches utilizing gene therapy, immunomodulation, and stem cell-based regeneration (Figure 19). While most remain investigational, these approaches directly target the pathophysiological mechanisms of RGC loss and hold promise for patients with disease progression despite IOP control.

##### Excitotoxicity

Excitotoxicity has been investigated as a therapeutic target in glaucoma, but clinical translation has been inconsistent. Specifically, while memantine demonstrated neuroprotective effects in preclinical models, two Phase III trials in nearly 2300 patients with POAG showed no meaningful preservation of peripheral vision over four years [102,103]. This discrepancy highlights a key translational gap between experimental neuroprotection and measurable clinical benefit. Modified derivatives such as memantine nitrate are being explored to combine neuroprotective and IOP-lowering effects, but current support remains limited to animal studies [104]. Brimonidine has also shown neuroprotective effects in preclinical studies and was associated with less VF progression than timolol in the Low-pressure Glaucoma Treatment Study despite similar IOP reduction; however, its clinical use was limited by higher discontinuation rates due to adverse events [102,105,106,107,108,109]. Other agents, including homotaurine and berberine, have shown anti-excitotoxic effects in preclinical models, but they still lack robust human data. Future work should prioritize controlled clinical trials with standardized structural and functional endpoints to determine whether these agents provide meaningful neuroprotection in glaucoma.

##### Mitochondrial Dysfunction

Mitochondrial dysfunction is an active therapeutic target in glaucoma, with current strategies focused mainly on nicotinamide, pyruvate, and coenzyme Q10 [102,110]. Among these, nicotinamide has some of the strongest preclinical support: in a dose-dependent mouse study, oral supplementation reduced RGC loss and lowered glaucoma incidence [111]. Its clinical efficacy, however, remains unproven, and two ongoing Phase III trials are evaluating whether nicotinamide can slow VF progression in POAG with or without concurrent IOP-lowering therapy [112,113]. Pyruvate has also shown early translational potential. A Phase II trial of combined nicotinamide and pyruvate reported short-term visual function improvement in POAG patients, but the durability and independent contribution of pyruvate remain unclear [114]. By contrast, evidence for coenzyme Q10 in glaucoma remains largely preclinical, with current interest driven more by mechanistic rationale than definitive clinical benefit [110]. Future studies should prioritize longer-term trials with standardized structural and functional endpoints to determine whether these therapies provide durable neuroprotection and truly slow glaucomatous progression in humans.

##### Oxidative Stress

Oxidative stress is a relevant therapeutic target in glaucoma, and the main agents studied in this area include CoQ10, ginkgo biloba extract (GBE), and citicoline [102].

CoQ10 has been investigated for its antioxidant and mitochondrial effects, with most interest focused on topical delivery. In a prospective study, adjunctive topical CoQ10-vitamin E was associated with better preservation of VF function, less RGC layer thinning on OCT, and improved VEP responses compared with standard therapy alone despite similar IOP control [115]. These findings are encouraging, but current clinical support for CoQ10 remains limited and insufficient to establish a clear neuroprotective effect.

Evidence for GBE is mixed and appears strongest in patients with NTG. Quaranta et al. (2003) found significant VF improvements in NTG patients after one month of GBE treatment [116], while Guo et al. (2014) found no benefit using the same regimen [117]. However, Lee et al. (2013) demonstrated that long-term GBE treatment (≥4 years) significantly slowed VF progression in NTG patients [118]. Larger, well-designed trials are needed to clarify GBE’s role in glaucoma management.

Citicoline currently has some of the most encouraging clinical data among antioxidant-oriented therapies. A 2019 two-year study showed that oral citicoline as adjunctive therapy to IOP-lowering medications significantly preserved RNFL and GCC thickness compared with IOP-lowering therapy alone, with benefits maintained through 24 months [119]. A 4-month study found that combining citicoline with homotaurine and vitamin E improved contrast sensitivity and quality of life in POAG patients [120]. Ongoing trials are further evaluating citicoline’s role in glaucoma, including studies examining oral citicoline’s effects on post-retinal neural conduction [121] and topical citicoline eye drops for slowing VF progression [122]. Taken together, these studies suggest that citicoline is a promising adjunctive candidate for glaucoma neuroprotection, but current evidence remains limited and insufficient to support routine clinical use.

Other antioxidants, including alpha-luminol, resveratrol, stanniocalcin-1, and alpha-lipoic acid, remain supported primarily by preclinical studies [123]. Their relevance to glaucoma is therefore still preliminary, and further work is needed to determine whether any of these agents can achieve clinically meaningful RGC protection in humans.

#### 3.7.2. Axonal Transport Disruption & Neurotrophic Deprivation

Neurotrophic deprivation remains a plausible therapeutic target in glaucoma, but the supporting evidence is still weighted heavily toward preclinical studies. Among approved glaucoma therapies, brimonidine is notable because preclinical data suggest it may increase BDNF expression in RGCs [106].

Over the past two decades, several preclinical studies have tested strategies to counter neurotrophic factor deprivation by artificially restoring or augmenting BDNF-TrkB signaling in glaucomatous or OHT animal models. For instance, an early rat glaucoma model demonstrated that overexpression of the BDNF gene significantly increased RGC survival as assessed by axon counts [124]. Beyond exogenous NTF supplementation, targeting BDNF receptors and other negative regulators of NTFs, such as LINGO-1, may also enhance RGC survival. In a chronic OHT rat model, simultaneous administration of BDNF and a soluble LINGO-1 receptor antagonist almost completely prevented RGC death over the study period [125]. This research highlights a promising avenue for future therapies, which will involve targeting both NTFs and their receptors/negative regulators to enhance their effects on RGC survival.

#### 3.7.3. Gene Therapy Approaches

Although neurotrophic factor delivery remains the most widely studied gene-based approach for RGC protection, several other targets have also shown benefit in experimental models. AAV-mediated suppression of PTEN or SOCS3 and modulation of KLF transcription factors have been reported to promote axonal regeneration and improve RGC survival after optic nerve injury [126]. Other strategies, including overexpression of NMNAT isoforms or OPA1, have been associated with preservation of axonal structure and greater resistance to glaucomatous stress [127]. Gene-based inhibition of apoptotic signaling and modulation of neuroinflammatory pathways, including viral delivery of TNF-α inhibitors, have also demonstrated neuroprotective effects [126]. Taken together, these targets represent emerging experimental avenues for gene-based RGC protection.

#### 3.7.4. Immunomodulation & Anti-Inflammatory Strategies

Immunomodulatory strategies in glaucoma have focused on cytokine signaling, glial reactivity, complement activation, and intracellular inflammatory pathways. Preclinical studies support cytokine modulation as one potential approach to limiting secondary RGC injury. In a rat glaucoma model, TNF-α inhibition with etanercept reduced RGC apoptosis and preserved optic nerve integrity [128]. IL-1 blockade has likewise been associated with reduced inflammatory signaling and improved neuronal survival in experimental settings.

Glial modulation has also shown neuroprotective effects in experimental glaucoma. Minocycline, which suppresses microglial activation and inflammatory cytokine production, has been associated with reduced inflammatory signaling and improved RGC survival across multiple preclinical models [129].

Complement inhibition represents another targeted strategy. In a rat model, C5-deficient animals developed less severe glaucoma and showed slower disease progression [130]. Interest in this pathway is supported further by the clinical use of complement inhibitors in other diseases, although their role in glaucoma has not yet been established [131].

Other studies have examined more selective immunomodulatory approaches, including inhibition of JAK/STAT and inflammasome-related signaling, with reported reductions in neuroinflammatory markers and preservation of RGC structure in experimental models [132]. Together, these findings support inflammation as a relevant therapeutic target, but the most effective pathway for clinical translation remains unclear.

#### 3.7.5. Stem Cell & Regenerative Therapies

Stem cell-based strategies in glaucoma have been explored both for direct RGC replacement and for support of surviving neurons. Direct replacement remains difficult because transplanted RGCs must survive, integrate into the retina, and extend axons through the optic nerve to central targets. Although protocols using embryonic stem cells and induced pluripotent stem cells can generate RGC-like cells that express canonical markers and show axon-like outgrowth in vitro [133,134], functional integration after transplantation remains limited [135].

By contrast, the supportive role of stem cells appears more immediately relevant. Mesenchymal stem cells and neural progenitor cells have been shown in experimental glaucoma models to secrete neurotrophic and immunomodulatory factors that enhance endogenous RGC survival and reduce inflammatory injury [136,137]. Stem cell-derived retinal organoids and RGC cultures have also become useful research platforms for studying human RGC biology and testing candidate therapies [138,139]. These findings support stem cell-based strategies as potential future avenues for neuroprotection, disease modeling, and eventual RGC replacement in glaucoma. However, significant barriers such as limited integration of transplanted cells, challenges in axonal guidance to central targets, and uncertain long-term safety restrict clinical translation. As such, stem cell therapies remain in experimental and pre-clinical stages.

#### 3.7.6. Novel Drug Delivery Systems & Nanomedicine

Nanomedicine is being investigated in glaucoma primarily as a drug delivery strategy rather than as a therapeutic mechanism itself. Its relevance lies in improving ocular penetration, prolonging drug residence, and increasing delivery to sites involved in RGC degeneration [140].

Among the systems studied, polymeric nanoparticles such as PLGA- and chitosan-based carriers have been used to deliver antioxidants, calcium-channel blockers, and anti-excitotoxic agents [141]. In animal models, nanoparticle-mediated delivery has been associated with reduced apoptosis compared with equivalent free-drug formulations [142,143]. These findings suggest that nanocarriers may enhance the effectiveness of neuroprotective agents, although the evidence remains largely experimental.

Nanomedicine has also been applied to neurotrophic factor delivery. Liposomes, dendrimers, and nanoparticles are being used to improve the stability and delivery of BDNF, CNTF, and NGF, which otherwise have poor ocular penetration and short half-lives [144]. In experimental settings, these approaches have been associated with reduced dendritic and axonal degeneration after ocular hypertensive injury [144]. Collectively, these approaches underscore the growing role of advanced delivery systems in glaucoma research.

#### 3.7.7. Glucagon-like Peptide-1 Receptor Agonists (GLP-1 RAs)

GLP-1 receptor agonists, originally developed for type 2 diabetes and obesity, have recently drawn interest in glaucoma because of their reported anti-inflammatory and neuroprotective effects. GLP-1 receptors are also expressed in multiple retinal layers, including the ganglion cell layer, which supports their biologic relevance to RGC injury [145].

Clinical interest has been driven mainly by observational data. In a large TriNetX cohort study, GLP-1 RA use was associated with a lower risk of developing POAG compared with several other common medications (insulin, metformin, and aspirin) [146]. Similar findings were reported in a meta-analysis of more than 156,000 diabetic patients, which found reduced glaucoma incidence among GLP-1 RA users compared with those receiving other antidiabetic therapies, including metformin and SGLT2 inhibitors [147]. These findings are notable, but they address glaucoma incidence rather than direct preservation of RGC structure or function.

## 4. Discussion

As highlighted in this review, RGC death in glaucoma arises from the convergence of mechanical stress, vascular insufficiency, metabolic dysfunction, and inflammatory signaling. Advances in OCT have transformed early detection of structural RGC compromise, creating an opportunity for intervention before irreversible functional loss. These tools not only refine risk stratification but also provide objective biomarkers for monitoring progression and therapeutic response.

Rather than representing independent pathways, the mechanisms underlying RGC degeneration appear to function as an interconnected network of injury. Mechanical stress at the LC, vascular insufficiency, and metabolic dysfunction converge to disrupt axonal transport, promote oxidative stress, and activate neuroinflammatory cascades, ultimately leading to apoptotic cell death. This integrated model suggests that glaucomatous neurodegeneration is not driven by a single dominant pathway but rather by the cumulative effects of multiple interacting processes, which may vary across patients and disease stages.

Despite effective IOP-lowering strategies, continued progression in many patients underscores the need for mechanism-based interventions. Emerging neuroprotective therapies addressing excitotoxicity, oxidative stress, mitochondrial dysfunction, and neurotrophic insufficiency reflect an evolving approach that extends beyond pressure reduction alone. Additionally, regenerative approaches, including stem cell and gene-based therapies, offer the potential to preserve or restore RGC integrity.

Importantly, the strength of evidence supporting these mechanisms and therapeutic strategies varies considerably. While pathways such as oxidative stress, mitochondrial dysfunction, and apoptosis are supported by robust experimental data, clinical validation remains limited for many proposed interventions. Similarly, emerging imaging and electrophysiologic biomarkers demonstrate promising associations with disease severity, but variability in methodology and lack of standardization limit their immediate clinical applicability. These discrepancies highlight the need to interpret findings within the context of study design and level of evidence.

The evidence included in this review is heterogeneous, encompassing clinical, preclinical, and basic science studies with varying methodologies and outcome measures. Many studies are derived from animal or in vitro models, which may limit direct clinical translation but do provide new avenues for investigation in the future. Additionally, variability in imaging techniques, biomarker definitions, and therapeutic approaches across studies introduces challenges in direct comparison and synthesis of findings. With regards to methodological limitations, formal quantitative synthesis and risk-of-bias assessment were not performed due to the heterogeneity of included studies, and were not amenable to a single standardized tool. Lastly, the inclusion of only English-language publications may introduce selection bias.

Despite its limitations, this review highlights important clinical and research implications. Given that RGC degeneration lies at the core of glaucomatous vision loss, deeper investigation into RGC biology and cellular vulnerability is essential. Integrating molecular insights with advanced imaging and biomarker-driven endpoints may facilitate earlier detection and more individualized approaches to disease monitoring, although further validation is required before widespread clinical implementation.

Future research should focus on bridging the gap between mechanistic insights and clinically actionable biomarkers. Priorities include the development of standardized imaging protocols, validation of emerging modalities such as OCTA and DARC in large-scale clinical studies, and integration of multimodal data through AI to improve early detection and progression prediction. In addition, greater emphasis on longitudinal studies and translational research is needed to determine which mechanistic pathways represent viable therapeutic targets. Addressing these challenges will be critical for advancing beyond the current IOP-centric management toward comprehensive, mechanism-based treatment strategies.

## 5. Conclusions

This review underscores both the complexity of glaucomatous RGC degeneration and the growing need to move beyond an exclusively IOP-centered view of disease progression. A broad range of mechanistic pathways underlying RGC degeneration and therapeutic strategies have been investigated, including approaches targeting excitotoxicity, oxidative stress, mitochondrial dysfunction, neurotrophic insufficiency, inflammation, and regenerative repair. Advances in OCT(A) and related imaging modalities have also expanded the ability to detect early structural injury and may support more refined disease monitoring and therapeutic assessment.

Overall, the current literature reflects strong mechanistic promise but uneven clinical translation. Many proposed neuroprotective and regenerative approaches are supported by experimental and preclinical data, yet comparatively few have demonstrated clear and durable benefit in human disease. Future progress will require standardized biomarkers, longitudinal validation, and adequately powered translational and clinical studies to determine which strategies can meaningfully preserve RGC structure and function.

## Figures and Tables

**Table 1:** PICOS criteria for inclusion of studies.

Parameter	Description
Population	Human and/or animal studies that involve RGC structure, function, degeneration, or therapeutic targeting in the context of glaucoma or related optic neuropathies.
Intervention	Experimental/therapeutic agents used for inhibition of RGC cell death or promoting regeneration.
Comparison	RGC features before and after interventions.
Outcomes	RGC cell death or apoptosis, nerve fiber layer thickness, glutamate concentration, inflammatory markers, and mitochondrial cytochrome c levels.
Study Design	Randomized/non-randomized, controlled/uncontrolled.

**Abbreviations**: RGC: retinal ganglion cell.

**Table 2:** Retinal ganglion cell subtypes and their role in visual processing.

RGC Subtype	Function
Midget	P pathway, spatial detail & red/green color contrast
Parasol	M Pathway, light contrast & motion
Small Bistratified	K pathway, blue/yellow color opponency
Smooth Monostratified	Light detection & motion sensitivity
ipRGCs	Circadian photoentrainment, light reflexes, sleep–wake regulation

**Abbreviations**: P: parvocellular; M: magnocellular; K: koniocellular; ipRGCs: intrinsically photosensitive retinal ganglion cells.

**Table 3:** Alternative apoptosis processes.

Cell Death Mechanism	Distinct Key Point(s)	Molecular Mediators	Role in RGC Dysfunction
Necroptosis [33] Ensures cell death when apoptosis is blocked (pathogens or mutations)	Fallback regulated cell death pathway	RIPK1, RIPK3, MLKL	When apoptosis is inhibited (impaired caspase activation, mitochondrial signaling), RGCs may switch to necroptosis, ensuring RGC death
Pyroptosis [33] Alerts other cells of stress conditions, release cytokines, and recruit immune cells	Intense inflammatory cell death to alert nearby cells	Caspase-1, Gasdermin D	Triggered by inflammatory molecules which amplify neuroinflammation, causing secondary damage to nearby RGCs
Ferroptosis [34] Removes damaged, injured, or abnormal cells	Toxic metabolic-oxidative cell death, via lipid membrane destruction	Iron, Lipid peroxides	Triggered by failure of antioxidant defense systems, inducing iron-driven lipid molecules that overwhelm the cells
Autophagy [35] Recycles and cleans damaged proteins and organelles to maintain cell health	Excessive autophagy and results in “self-digestion” and cell death	Beclin-1, LC3, ATG proteins	Typically acts to recycle and clean up damaged proteins and organelles triggered by undue stress (e.g., glaucoma)

**Abbreviations**: RGC: retinal ganglion cell. RIPK1: receptor-interacting protein kinase 1; RIPK3: receptor-interacting protein kinase 3; MLKL: mixed lineage kinase domain-like protein; LC3: microtubule-associated protein 1 light chain 3; ATG: autophagy-related proteins.

**Table 4:** Comparative Features of Glaucoma and Other Optic Neuropathies.

Disease	Underlying Pathology	Clinical/Fundus Findings	Patterns of Nerve Fiber Loss/OCT Findings	Distinguishing Features
Glaucoma [1,39]	Chronic mechanical and/or vascular RGC injury	Progressive vertical optic disc cupping, neuroretinal rim thinning	Superotemporal/inferotemporal notching, RNFL and vessel density loss	Gradual peripheral vision loss, arcuate scotomas, nasal step, central island
ION [39]	NAION: noninflammatory ONH hypoperfusion, AAION: inflammatory occlusion of posterior ciliary arteries, usually from giant cell arteritis	Acute optic disc swelling with flame hemorrhages, ONH pallor	Superonasal thinning, rim relatively preserved early on, sectoral or altitudinal RNFL loss	Acute painless vision loss, dense altitudinal defect (AAION)
Optic Neuritis [40,41]	Acute inflammatory demyelination of optic nerve	Pain with eye movement, optic disc swelling may evolve to pallor	Early RNFL thickening, then temporal pRNFL and macular GCIPL loss	Rapid vision loss, central scotoma
NMOSD [42,43,44]	AQP4-IgG astrocytopathy with complement-mediated injury	Severe, often bilateral optic neuritis, relapsing attacks	Marked pRNFL and macular GCIPL thinning	Relapsing tendency than typical optic neuritis
MOGAD [44]	MOG-IgG autoimmune demyelination	Often unilateral optic neuritis, tends to recover better than NMOSD	pRNFL and macular GCIPL thinning similar to NMOSD	Better visual recovery than NMOSD
LHON [45,46]	Mitochondrial-based optic neuropathy due to mtDNA mutations	Early hyperemic disc with peripapillary telangiectatic vessels, ending in optic pallor	Early inferior/temporal thickening, followed by temporal pRNFL and macular GCIPL thinning of the papillomacular bundle	Central vision loss, dyschromatopsia, central or cecocentral scotoma
DOA [47,48]	OPA1-related mitochondrial optic neuropathy	Temporal optic disc pallor	Early temporal pRNFL and macular GCIPL thinning of the papillomacular bundle	Slowly progressive bilateral central vision loss, dyschromatopsia
CON [49]	ON/chiasm compression by mass lesion	Segmental or diffuse optic pallor	Macular GCIPL and pRNFL thinning based on compression site, often nasal GCIPL thinning	Reduced acuity, dyschromatopsia, central, cecocentral, hemianopic, or bitemporal defects

**Abbreviations**: OCT: optical coherence tomography; RNFL: retinal nerve fiber layer; RGC: retinal ganglion cell; ION: ischemic optic neuropathy; NAION: non-arteritic anterior ischemic optic neuropathy; ONH: optic nerve head; AAION: arteritic anterior ischemic optic neuropathy; pRNFL: peripapillary retinal nerve fiber layer; GCIPL: ganglion cell-inner plexiform layer; NMOSD: neuromyelitis optica spectrum disorder; AQP4-IgG: aquaporin-4 immunoglobulin G; MOGAD: myelin oligodendrocyte glycoprotein antibody-associated disease; MOG-IgG: myelin oligodendrocyte glycoprotein immunoglobulin G; LHON: Leber hereditary optic neuropathy; mtDNA: mitochondrial DNA; DOA: dominant optic atrophy; OPA1: optic atrophy 1; CON: compressive optic neuropathy; ON: optic nerve.

## Data Availability

No new data were created or analyzed during this study. Data sharing is not applicable to this article.
